# Ginger phytochemical corona enhances hemocompatibility of metal oxide nanoparticles for blood-contacting applications

**DOI:** 10.1038/s41598-026-50697-7

**Published:** 2026-05-09

**Authors:** Alaa Hassan Said, A. A. Ebnalwaled, Mary Samir, G. A. Gamal

**Affiliations:** 1 Electronic and Nano Devises Lab, Faculty of Science, South Valley University, Qena, 83523 Egypt; 2 Physics Department, Faculty of Science, South Valley University, Qena, 83523 Egypt

**Keywords:** Metal oxide nanoparticles, Green synthesis, Ginger, Hemocompatibility, Protein corona, Reactive oxygen species, Biochemistry, Biological techniques, Biotechnology, Chemistry, Drug discovery, Materials science, Nanoscience and technology

## Abstract

The clinical translation of metal oxide nanoparticles is critically dependent on their hemocompatibility. While chemical synthesis often yields reactive nanoparticles that adversely interact with blood components, green synthesis using plant extracts offers a promising alternative. This study compares the hemocompatibility of four metal oxide nanoparticles (TiO_2_, ZnO, MgO, and CaO) synthesized via conventional chemical precipitation and green synthesis using ginger (*Zingiber officinale*) extract. Ginger-mediated metal oxide nanoparticles formed a stable phytochemical corona, confirmed by FTIR and total phenolic content analysis (106.4 mg GAE/g in extract; 10.6–53.2 mg GAE/g bound to GMONPs). Dynamic light scattering revealed dramatically thinner protein coronas on green metal oxide nanoparticles (hard corona: 10–14 nm vs. 30–97 nm for chemical metal oxide nanoparticles), with ZnO-Ginger showing an 88% reduction (*p* < 0.001). SDS-PAGE demonstrated selective apolipoprotein A-I enrichment (3- to 8-fold) and reduced opsonin adsorption (87–91%) on green metal oxide nanoparticles. ROS quantification confirmed 55–81% reduction in oxidative stress, strongly correlating with hemolysis and PBMC viability. Green metal oxide nanoparticles were consistently non-hemolytic (< 2% vs. 8–18% for chemical metal oxide nanoparticles), preserved normal RBC morphology (> 92% discocytes), maintained PBMC viability > 93% at 500 µg/mL (vs. 68–78%), and showed neutral coagulation for ZnO-Ginger and MgO-Ginger. The safe concentration window expanded from < 125 µg/mL (chemical) to > 500 µg/mL (green). ZnO-Ginger NPs showed the most dramatic improvement: 88% corona reduction, 81% ROS reduction, 93% hemolysis reduction (18% → 1.2%), and > 2.7× IC_50_ increase (185 → >500 µg/mL), attributed to the highest phenolic loading (53.2 mg GAE/g) and ApoA-I enrichment (7.8-fold). Ginger extract is a highly effective green agent for producing hemocompatible metal oxide nanoparticles through four protective mechanisms: physical barrier, ion chelation, ROS scavenging, and stealth corona formation. This approach supports their potential use in blood-contacting biomedical applications.

## Introduction

The burgeoning field of nanotechnology has positioned metal oxide nanoparticles (MONPs) at the forefront of innovation across diverse sectors, including medicine, catalysis, and environmental remediation. Their unique physicochemical properties, derived from their high surface-area-to-volume ratio and quantum effects, offer unparalleled advantages. In biomedicine, particularly, MONPs such as titanium dioxide (TiO_2_), zinc oxide (ZnO), magnesium oxide (MgO), and calcium oxide (CaO) have demonstrated immense potential as drug delivery vehicles, imaging agents, and antimicrobial therapeutics^1–4^. However, the conventional chemical synthesis of these NPs often involves hazardous reducing agents and generates toxic byproducts, raising significant environmental and safety concerns. This has prompted a paradigm shift towards sustainable and eco-friendly synthesis methodologies^5^.

Green synthesis, which utilizes plant extracts as reducing and stabilizing agents, has emerged as a superior alternative to conventional routes. This approach is not only cost-effective and energy-efficient but also eliminates the need for toxic chemicals. Plant metabolites like flavonoids, alkaloids, and terpenoids facilitate the bioreduction of metal salts into stable NPs while simultaneously functionalizing their surface, which can enhance biocompatibility and functionality^6^. Recent studies have demonstrated that surface modification of MONPs with natural polymers and bioactive compounds enhances their biological properties. For instance, SnO_2_-chitosan-d-carvone nanocomposites have shown promising antimicrobial and anticancer activities^7^, while NiO NPs modified with carboxymethyl cellulose and d-carvone exhibited enhanced antimicrobial and antioxidant properties^8^. Similarly, surface modification of CaO NPs with CMC/D-carvone has been reported to enhance anticancer and antimicrobial activities^9^. These findings support the approach of using natural bioactive compounds for NPs functionalization.

Ginger (*Zingiber officinale*), a widely used medicinal plant, is a rich source of bioactive compounds, including gingerols and shogaols, which possess potent antioxidants and anti-inflammatory properties^10^. Employing ginger extract for the synthesis of MONPs (GMONPs) is therefore anticipated to yield NPs with inherently bioactive surfaces, potentially offering superior biological performance compared to their chemically synthesized counterparts^11^.

Despite the promising applications of MONPs, their successful translation into clinical practice requires a thorough evaluation of their biosafety. When introduced into the body, NPs inevitably interact with blood components, making hemocompatibility (a material’s compatibility with blood) a critical aspect of their toxicological assessment^12^. Hemocompatible NPs should minimize adverse effects such as hemolysis (rupture of red blood cells), disruption of plasma coagulation pathways, and negative impacts on other hematological parameters. Additionally, cytotoxicity to blood cells, including peripheral blood mononuclear cells (PBMCs), is an important indicator of overall safety^13^. Although many studies have focused on the synthesis and antibacterial properties of various MONPs, there is a lack of systematic and comparative investigations into the hemocompatibility of green-synthesized versus chemically synthesized MONPs.

This study aims to bridge this knowledge gap by providing a comprehensive assessment of the hemocompatibility of a series of MONPs (TiO2, ZnO, MgO, and CaO) synthesized via two different routes: a conventional chemical precipitation method and a green approach using ginger extract. We hypothesize that the ginger-mediated GMONPs will exhibit superior hemocompatibility due to the capping action of natural biomolecules. The objectives of this work are threefold: (1) to synthesize and characterize both chemical and green MONPs; (2) to systematically evaluate their hemolytic activity, effects on coagulation times (PT and APTT), erythrocyte sedimentation rate (ESR), and overall hematological parameters; and (3) to assess their cytotoxicity against human RBCs using the MTT assay. The findings of this research will provide crucial insights into the blood compatibility of ginger-synthesized NPs, informing their safe design for potential biomedical applications.

## Materials and methods

### Chemicals and reagents

All chemicals and reagents were of analytical grade and used without further purification. The precursor salts for nanoparticle synthesis included titanium(IV) isopropoxide (TTIP, ≥ 97%), zinc nitrate hexahydrate (Zn(NO_3_)_3_·6 H₂O, ≥ 99%), magnesium nitrate hexahydrate (Mg(NO_3_)_2_·6H_2_O, ≥ 99%), and calcium nitrate tetrahydrate (Ca(NO_3_)_₂_·4 H₂O, ≥ 99%), all purchased from Merck KGaA (Darmstadt, Germany). Sodium hydroxide (NaOH, ≥ 98%) and ethanol (≥ 99.8%) were obtained from Samchun Chemicals (South Korea). Fresh ginger (*Zingiber officinale*) rhizomes were procured from a local commercial market.

For cell culture and hemocompatibility assays, the following materials were purchased from Himedia (India): RPMI-1640 medium, Dulbecco’s Modified Eagle’s Medium (DMEM), fetal bovine serum (FBS), phosphate-buffered saline (PBS, pH 7.4), 0.05% Trypsin-EDTA, l-glutamine, penicillin–streptomycin solution (10,000 U/mL), trypan blue solution (0.4%), MTT reagent (3-(4,5-dimethylthiazol-2-yl)-2,5-diphenyltetrazolium bromide), and dimethyl sulfoxide (DMSO). Ficoll-Paque PLUS was obtained from Cytiva (USA). For protein corona analysis, human plasma was pooled from five healthy donors. All consumables (96-well cell culture plates, Eppendorf tubes, Falcon tubes, cell culture flasks) were supplied by Sarstedt (Germany).

#### Preparation of ginger rhizome aqueous extract

**Fig. 1:**
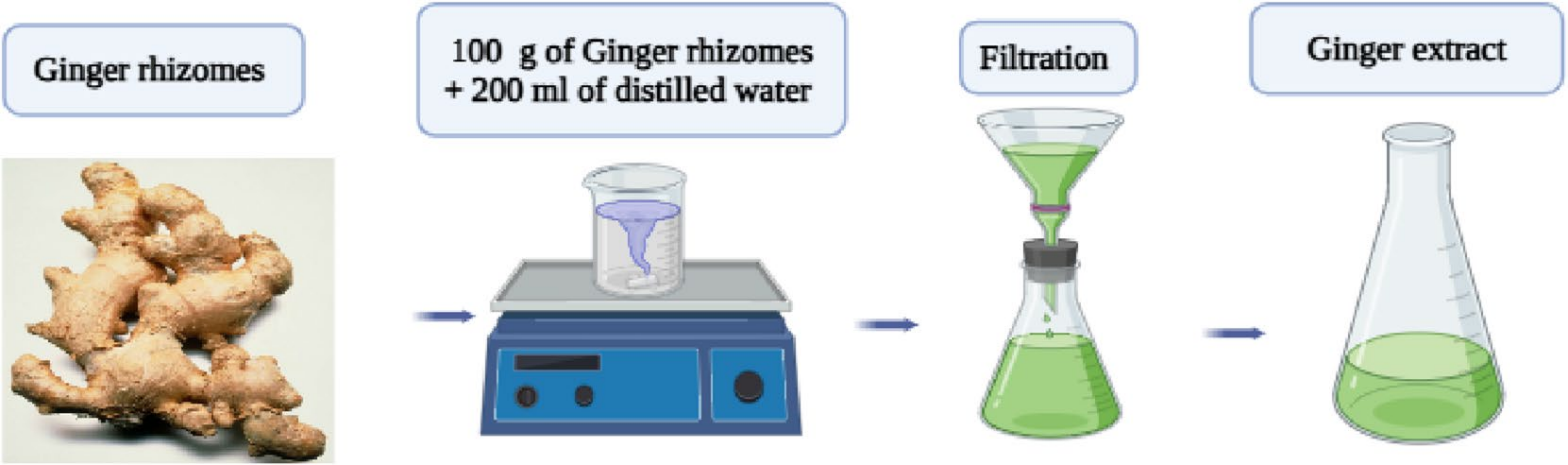
Preparation of aqueous extract of ginger.

Fresh ginger (*Zingiber officinale*) rhizomes were procured from a local market. To remove surface impurities, the rhizomes were thoroughly washed with distilled water, peeled, and thinly sliced. The slices were then air-dried at room temperature for 24 h^14^. Subsequently, 50 g of the dried ginger was added to 200 mL of distilled water and heated at 60–70 °C for 45 min under continuous stirring. The resulting mixture was cooled to room temperature and filtered through Whatman No. 1 filter paper. The clear filtrate, serving as the source of reducing and stabilizing biomolecules, was stored at 4 °C for further use in NPs synthesis as shown in Fig. 1^15^.

#### Nanoparticle synthesis

**Fig. 2:**
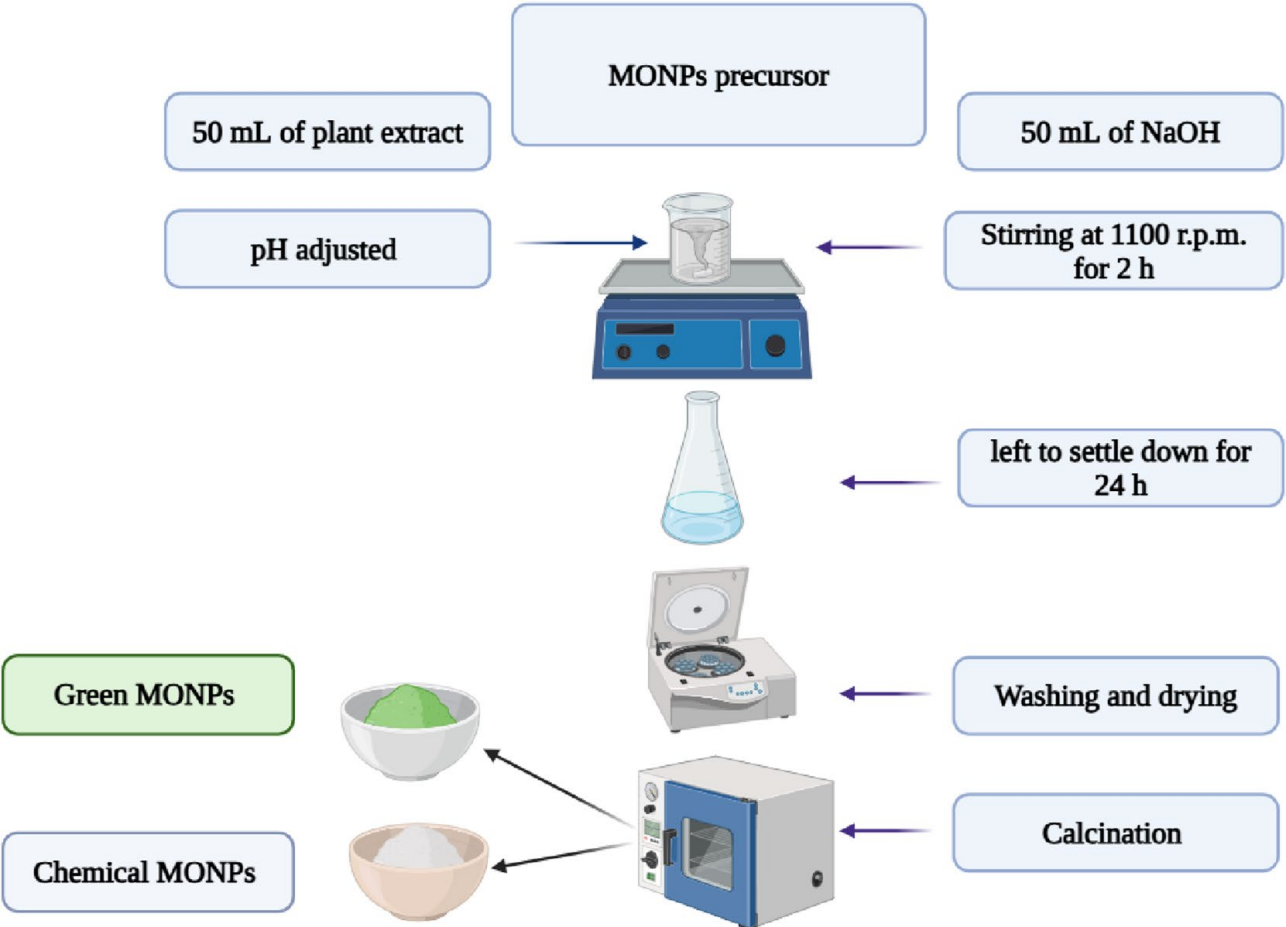
Schematic illustration of synthesis of MONPs.

A chemical precipitation method was employed to synthesize two sets of NPs for each metal oxide: one using traditional sodium hydroxide (denoted as MONPs) and another using ginger extract as a green reducing and capping agent (denoted as GMONPs)^16–19^. The synthesis was standardized as follows: for titanium oxide NPs (TiO_2_ and TiO_2_- Ginger NPs), the precursor consisted of 20 mL of titanium(IV) isopropoxide (TTIP) in 100 mL of distilled water. For zinc, magnesium, and calcium oxides (ZnO, MgO, CaO and their green counterparts), the precursor solutions were prepared by dissolving 5 g of the respective nitrate salts (Zn(NO_3_)_2_·6H_2_O, Mg(NO_2_)_2_, or Ca(NO_3_)_2_·4H_2_O) in 100 mL of distilled water. In each case, 50 mL of the reducing agent solution (either 2 M sodium hydroxide for conventional MONPs or the aqueous ginger extract for GMONPs) was added dropwise to the precursor solution under constant stirring at 1100 rpm for 1 h. The reaction pH was critical and was adjusted to 10 for the synthesis of Ti and Zn-based NPs, and to 12 for Mg and Ca-based NPs, using 2 M NaOH. The resultant precipitate was aged for 24 h, centrifuged, and sequentially washed with distilled water and ethanol. The purified pellets were dried in an oven at 100 °C for 8 h, manually ground into a fine powder, and finally calcined in a muffle furnace (450 °C for 2 h for TiO_2_, ZnO, and MgO; 650 °C for 3 h for CaO), as illustrated in Fig. 2.

### Physicochemical characterization

The crystalline structure of the synthesized NPs was analyzed using X-ray Diffraction (XRD, PANalytical X’Pert PRO). Morphological features were examined by High-Resolution Transmission Electron Microscopy (HR-TEM, JEOL JEM-2100). Surface functional groups were identified using Fourier-Transform Infrared (FTIR) spectroscopy (Jasco Model 6100). A UV-Vis spectrophotometer (Analytik Jena SPECORD 200 PLUS) was used to confirm NPs formation and to assess their colloidal stability in phosphate-buffered saline (PBS, pH 7.4) over 720 min. Hydrodynamic diameter and polydispersity index (PdI) were measured using a Zetasizer Nano ZS (Malvern Panalytical, UK) equipped with a 633 nm He-Ne laser at a scattering angle of 173°. Measurements were performed at 25 °C after 120 s of equilibration. Samples were diluted 1:10 with 10 mM sodium phosphate buffer (pH 7.4) containing 10 mM NaCl. Zeta potential was measured using folded capillary cells (DTS1070, Malvern). Each measurement consisted of 10–15 runs, and three independent measurements were performed per sample.

### Total phenolic content (TPC)

Total phenolic content was determined using the Folin-Ciocalteu method^20^. Briefly, 0.5 mL of ginger extract (appropriately diluted) was mixed with 2.5 mL of Folin-Ciocalteu reagent (diluted 1:10 with distilled water). After 5 min of incubation at room temperature, 2.0 mL of sodium carbonate solution (7.5% w/v) was added. The mixture was incubated for 60 min in the dark at room temperature, and absorbance was measured at 765 nm using a UV-Vis spectrophotometer (Analytik Jena SPECORD 200 PLUS). A standard calibration curve was prepared using gallic acid (0–200 µg/mL). TPC was expressed as milligrams of gallic acid equivalents per gram of dry extract (mg GAE/g). To quantify bound phenolics on GMONPs, 10 mg of dried NPs was suspended in 5 mL of 70% methanol (v/v) and sonicated for 30 min at 40 kHz, 25 °C to release bound phenolics. The suspension was centrifuged at 10,000×*g* for 10 min at 4 °C, and the supernatant was collected. The extraction was repeated twice, and the pooled supernatants were analyzed using the same protocol. Chemically synthesized MONPs were processed identically as negative controls. All measurements were performed in triplicate.

### Protein corona analysis

#### Protein corona formation

NPs (1 mg/mL) were incubated with 50% human plasma (pooled from five healthy donors) for 1 h at 37 °C with gentle shaking. For soft corona analysis, samples were diluted 1:10 with PBS and measured immediately by DLS. For hard corona analysis, NPs were centrifuged (16,000×*g*, 15 min, 4 °C) and washed three times with PBS^21^.

#### SDS-PAGE analysis

Bound proteins were eluted from hard corona samples by boiling in Laemmli buffer (95 °C, 10 min). Proteins were separated on 12% SDS-PAGE gel (120 V, 90 min) and visualized by Coomassie Blue R-250 staining. A prestained protein ladder (10–250 kDa) was used as molecular weight marker^22^. Densitometric analysis was performed using ImageJ software, with band intensities normalized to albumin in human plasma (set to 100%).

### Reactive oxygen species (ROS) quantification

ROS generation was evaluated using the DCFH-DA assay. RBCs (2% suspension) were treated with NPs (200 µg/mL) for 2 h, and PBMCs (1 × 10^5^ cells/well) were treated with NPs (1.95–500 µg/mL) for 24 h at 37 °C. DCFH-DA (20 µM) was added for the final 30 min of incubation. Fluorescence was measured at Ex/Em = 485/535 nm using a microplate reader. H₂O₂ (100 µM) served as positive control. Results were expressed as fold-change relative to untreated control^23^.

### Hemocompatibility assessment

#### Blood collection and ethical statement

Human blood samples were collected from five healthy, consenting adult volunteers following approval from the Institutional Ethics Committee (Permit Number: 001/12/24). All donors were non-smokers and had not taken any medication for two weeks prior to donation. Blood was drawn into vacuum tubes containing EDTA or sodium citrate as anticoagulants, as required for specific assays. All experiments were performed in triplicate for each donor, and results are presented as mean ± standard deviation.

#### Hemolysis assay

The hemolytic potential of the NPs was evaluated as per established protocols. Briefly, red blood cells (RBCs) were isolated from whole blood by centrifugation, washed with PBS (pH 7.4), and reconstituted as a 2% (v/v) suspension. Various concentrations of NPs suspensions (5–200 µg/mL) were incubated with the RBC suspension at 37 °C for 2 h. After incubation, the samples were centrifuged, and the absorbance of the supernatant was measured at 540 nm to quantify released hemoglobin. PBS and 1% Triton X-100 served as the negative (0% hemolysis) and positive (100% hemolysis) controls, respectively. The hemolysis percentage was calculated as follows^24^:

1 $${\mathrm{Hemolysis}} \, (\% )=\frac{{{A_s} - {A_{NC}}}}{{{A_{PC}} - {A_{NC}}}} \times 100$$

where A_s_ is the absorbance of test samples, A_NC_ is the absorbance of negative control and A_PC_ density is the absorbance of positive control of control.

#### Erythrocyte sedimentation rate (ESR)

The influence of NPs on the erythrocyte sedimentation rate was determined using the Westergren method. Whole blood was incubated with NPs (2.5–100 µg/mL) for 3 h at room temperature. The mixture was then aspirated into Westergren tubes and placed vertically^25^. The distance the RBC column fell after 1 h was recorded as the ESR value in mm/h.

#### Coagulation profile

The effect on plasma coagulation was investigated by measuring Prothrombin Time (PT) and Activated Partial Thromboplastin Time (APTT)^26^. Platelet-poor plasma was incubated with NPs (100 µg/mL) for 30 min at 37 °C. For PT, thromboplastin reagent was added to the plasma, and the time to clot formation was recorded. For APTT, plasma was first mixed with APTT reagent and then recalcified with CaCl_2_, and the clotting time was measured.

#### In vitro cytotoxicity (MTT assay)

The cytotoxicity of the MONPs on human peripheral blood mononuclear cells (PBMCs) was evaluated using the MTT assay^27^. Human PBMCs were isolated from fresh EDTA-anticoagulated whole blood by density-gradient centrifugation. Briefly, 5 mL of whole blood was carefully layered over 5 mL of Ficoll-Paque PLUS in a 15 mL centrifuge tube and centrifuged at 400×*g* for 30 min at 20 °C with the brake off. The opaque interface containing PBMCs was carefully transferred to a new tube, washed twice with PBS, and centrifuged at 250×*g* for 10 min. The cell pellet was resuspended in RPMI-1640 medium supplemented with 10% FBS, 2 mM L-glutamine, and 1% penicillin–streptomycin. Cell viability was assessed by trypan blue exclusion, and only preparations with > 95% viability were used for experiments.

For the MTT assay, isolated PBMCs were seeded in 96-well tissue culture plates at a density of 1 × 10^5^ cells/well in 100 µL of complete RPMI-1640 medium and incubated for 24 h at 37 °C in a humidified 5% CO_2_ atmosphere. Following incubation, the medium was replaced with fresh medium containing serial dilutions of nanoparticle suspensions (1.95–500 µg/mL) prepared in sterile PBS. Cells treated with complete medium alone served as the negative control (100% viability), while cells treated with 10% DMSO served as the positive control (0% viability). Each concentration was tested in triplicate, and the experiment was repeated independently three times. After 24 h treatment, the medium was replaced with fresh medium containing MTT (0.5 mg/mL) and incubated for another 4 h to allow formazan crystal formation. The crystals were dissolved in DMSO, and absorbance was measured at 570 nm using a microplate reader. Cell viability was calculated as a percentage relative to the untreated negative control using the following formula^28^:

2 $${\text{Cell viability}} \, (\% )=\frac{{{A_{570{\mathrm{(sample)}}}} - {A_{630{\mathrm{(sample)}}}}}}{{{A_{570{\mathrm{(control)}}}} - {A_{630{\mathrm{(control)}}}}}} \times 100$$

where (A)_sample_ is the absorption of the test sample and (A)_control_ is the absorption of the control at 570 nm. Data are presented as mean ± SD of three independent experiments performed in triplicate. Half-maximal inhibitory concentration (IC_50_) values were calculated by non-linear regression analysis using a four-parameter logistic curve.

#### Analysis of hematological parameters

To assess the impact on blood cells, whole blood was incubated with a high concentration of NPs (2 mg/mL) for 3 h. The concentration ranges used in this study were selected based on clinical relevance and safety margin assessment. The lower range (5–200 µg/mL) brackets the peak plasma concentrations of FDA-approved nanomedicines (e.g., Doxil^®^ ~50–100 µg/mL)^29^. The intermediate range (200–500 µg/mL) simulates local nanoparticle accumulation in tissues via the enhanced permeability and retention (EPR) effect^30^. The highest concentrations (500 µg/mL for PBMCs, 2 mg/mL for hematological parameters) serve as worst-case stress tests to establish safety margins and identify potential toxicity mechanisms, consistent with standard nanotoxicology practice^31^.

Post-incubation, the following parameters were analyzed manually or spectrophotometrically: total Red Blood Cell (RBC) and White Blood Cell (WBC) counts using a hemocytometer, hemoglobin (Hb) concentration, and hematocrit (Hct)^32^. These values were used to calculate hematological indices: Mean Corpuscular Volume (MCV), Mean Corpuscular Hemoglobin (MCH), and Mean Corpuscular Hemoglobin Concentration (MCHC). A differential leukocyte count was also performed.

### Statistical analysis

All data are expressed as mean ± standard deviation (SD) from at least three independent experiments. Statistical significance was determined using one-way analysis of variance (ANOVA) followed by Tukey’s post-hoc test for multiple comparisons or Student’s t-test for two-group comparisons. A p-value of less than 0.05 was considered statistically significant. Correlations were analyzed using Pearson’s correlation coefficient.

Inter-donor variability was assessed by calculating the coefficient of variation (CV%) for each assay across the five donors using the formula: CV% = (SD/mean) × 100. Effect sizes for key comparisons (chemical vs. green NPs at 200 µg/mL) were calculated using Cohen’s d, defined as (mean_1_ − mean_2_)/pooled SD, with values of 0.2 considered small, 0.5 medium, and 0.8 large effects^33^. All statistical analyses were performed using GraphPad Prism 9.0 (GraphPad Software, San Diego, CA, USA).

## Results and discussion

### Characterization of MONPs

#### X-ray diffraction (XRD)

**Fig. 3:**
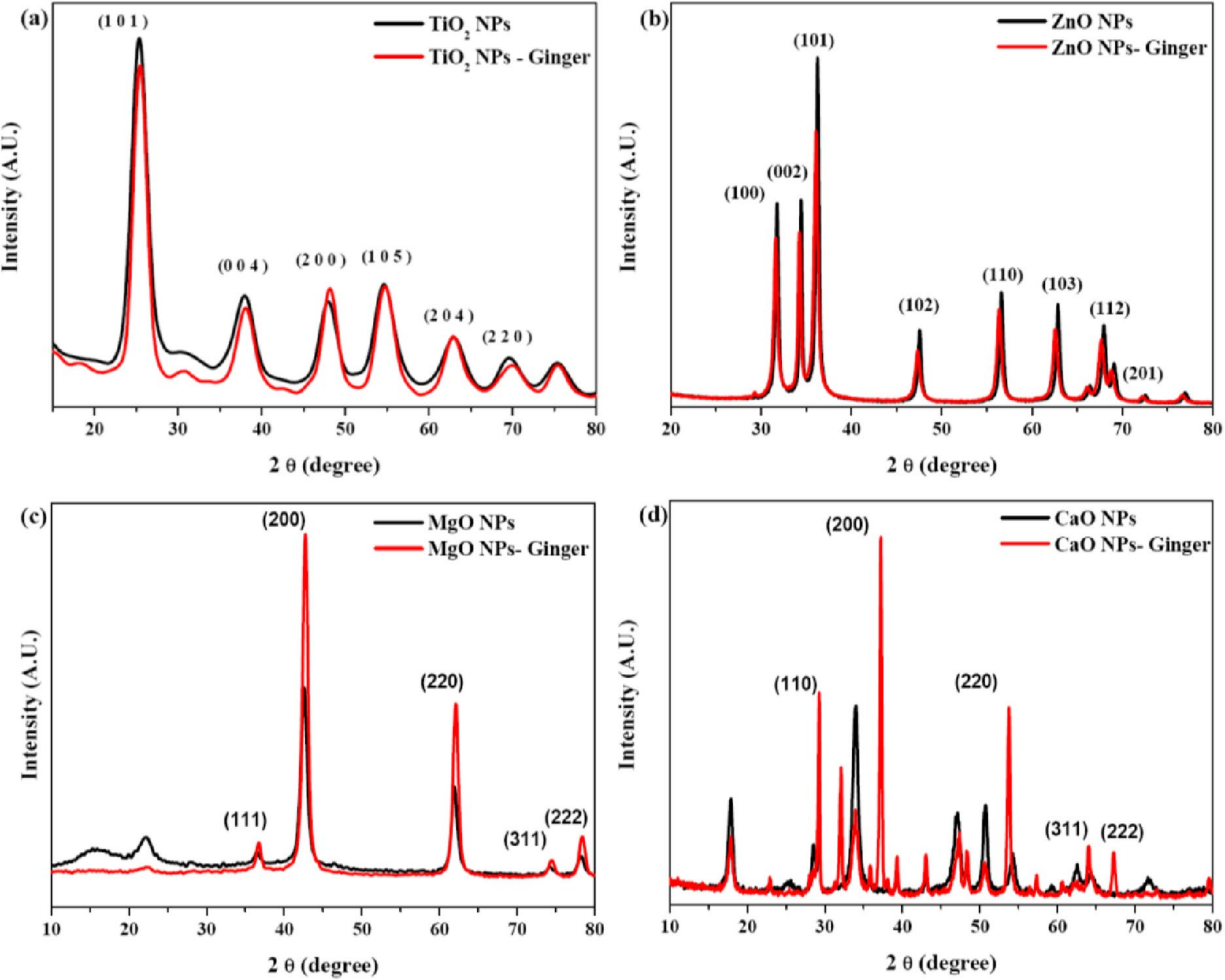
XRD patterns of MONPs and their ginger mediated GMONPs. (**a**) TiO_2_ NPs, (**b**) ZnO NPs, (**c**) MgO NPs and (**d**) CaO NPs.

The successful synthesis and crystalline nature of the MONPs were confirmed using X-ray diffraction (XRD) analysis. Figure 3 illustrates the comparative XRD patterns of chemically synthesized and ginger-mediated NPs for the four investigated metal oxides: (a) TiO_2_, (b) ZnO, (c) MgO, and (d) CaO. All diffraction patterns exhibit sharp and well-defined peaks, indicating the high crystallinity of the synthesized materials.

The diffraction peaks of the chemically synthesized NPs were successfully indexed to their respective standard crystal structures: anatase TiO_2_ (JCPDS No. 21-1272), hexagonal wurtzite ZnO (JCPDS No. 36-1451), cubic periclase MgO (JCPDS No. 45-0946), and cubic rocksalt CaO (JCPDS No. 37-1497)^34–37^. Notably, the XRD patterns of the ginger-mediated MONPs closely matched those of their chemically synthesized counterparts, confirming that the use of ginger extract did not alter the fundamental crystal structure of the metal oxides. No additional impurity-related phases were detected for TiO_2_, ZnO, and MgO NPs, while minor low-intensity reflections observed in CaO samples are consistent with the known hygroscopic nature of CaO and its tendency toward partial surface hydration or carbonation.

These findings are consistent with previous reports on green synthesis using medicinal plant extracts, such as the formation of phase-pure anatase TiO_2_ using *Aloe vera*^38^ and wurtzite ZnO synthesized with *Cymbopogon citratus* (lemongrass) extract^39^. Natural polymers significantly influence the structural and optical properties of green-synthesized ZnO NPs^40^.

The average crystallite size (D) and dislocation density (δ) were estimated from the XRD data and are summarized in Table 1. As expected, an inverse relationship between crystallite size and dislocation density (δ = 1/D^2^) was observed across all samples. For TiO_2_ NPs, both synthesis routes yielded comparable crystallite sizes (20.03 nm for chemically synthesized vs. 19.74 nm for ginger-mediated) and corresponding dislocation densities (25.89 × 10^−4^ nm^−2^ and 26.97 × 10^−4^ nm^−2^, respectively). This similarity indicates that ginger-mediated synthesis effectively stabilizes TiO_2_ NPs without introducing excessive crystallographic defects.

**Table 1:** Crystallographic parameters of chemically synthesized and ginger-mediated MONPs.

Nanoparticle	Synthesis route	Average crystallite size, D (nm)	Dislocation density, δ (×10^−4^ nm^−2^)	Lattice constant (Å)	Unit cell volume (Å^3^)
TiO_2_	Chemical	20.03 ± 2.53	25.89 ± 6.05	a = b=3.790, c = 9.468	136.0
	Ginger	19.74 ± 2.95	26.97 ± 7.30	a = b=3.775, c = 9.438	134.5
ZnO	Chemical	21.47 ± 3.72	24.89 ± 8.24	a = b=3.247, c = 5.203	47.53
	Ginger	14.62 ± 3.69	53.80 ± 9.01	a = b=3.260, c = 5.232	48.16
MgO	Chemical	10.63 ± 0.75	89.65 ± 14.11	a = 4.204	74.31
	Ginger	11.49 ± 1.99	82.08 ± 12.14	a = 4.243	76.44
CaO	Chemical	13.17 ± 2.40	37.88 ± 11.13	a = 4.723	105.6
	Ginger	14.44 ± 4.11	63.24 ± 15.90	a = 4.776	109.1

High standard deviation reflects heterogeneity in crystallite size distribution.

A more pronounced effect of green synthesis was observed for ZnO NPs. Ginger-mediated ZnO exhibited a significantly reduced crystallite size (14.62 nm) compared to chemically synthesized ZnO (21.47 nm), accompanied by an increase in dislocation density from 24.89 × 10^−4^ nm^−2^ to 53.80 × 10^−4^ nm^−2^. The higher defect density suggests increased lattice distortion, which may enhance surface reactivity and influence subsequent biological interactions.

For MgO NPs, ginger mediation resulted in a slight increase in crystallite size (from 10.63 nm to 11.49 nm) and a corresponding decrease in dislocation density (from 89.65 × 10^−4^ nm^−2^ to 82.08 × 10^−4^ nm^−2^). This trend suggests that phytochemicals present in the ginger extract may promote more ordered crystal growth for MgO. In contrast, CaO NPs exhibited more complex behavior. Ginger-mediated CaO showed a modest increase in crystallite size (14.44 nm) compared to chemically synthesized CaO (13.17 nm), along with an increase in dislocation density (63.24 × 10^−4^ nm^−2^ vs. 37.88 × 10^−4^ nm^−2^. This apparent discrepancy suggests that although crystallite growth is promoted, ginger-mediated CaO NPs may contain higher internal strain or defect density, potentially arising from surface interactions with organic constituents or post-synthesis atmospheric exposure^41,42^.

Analysis of lattice parameters revealed subtle but significant modifications induced by the green synthesis route. For TiO_2_ NPs, ginger mediation caused a slight contraction of both the a-axis (from 3.790 Å to 3.775 Å) and c-axis (from 9.468 to 9.438 Å), resulting in a reduction in unit cell volume from 136.0 to 134.5 Å^3^. Such lattice contraction may be associated with the generation of oxygen vacancies or lattice strain induced during green synthesis^10^.

Conversely, ZnO NPs exhibited lattice expansion upon ginger mediation, with the a-axis increasing from 3.247 to 3.260 Å and the c-axis from 5.203 to 5.232 Å, leading to an increase in unit cell volume from 47.53 to 48.16 Å^3^. Similar lattice expansion has been widely reported for plant-mediated ZnO synthesis and is often attributed to intrinsic defects or the incorporation of organic species at the nanoparticle surface or near-surface lattice sites^11,43^.

Both MgO and CaO NPs also showed lattice expansion following ginger-mediated synthesis. For MgO, the lattice parameter increased from 4.204 to 4.243 Å, with the unit cell volume expanding from 74.31 Å to 76.44 Å^3^. Likewise, CaO exhibited an increase in lattice parameter from 4.723 to 4.776 Å and unit cell volume from 105.6 to 109.1 Å^3^. These expansions suggest the introduction of lattice strain or point defects, potentially arising from interactions between the metal oxide lattice and bioactive constituents of the ginger extract^44,45^.

Overall, the variation in crystallite size, defect density, and lattice parameters across different metal oxides highlights the metal-specific nature of ginger-mediated green synthesis. The phytochemicals present in the extract interact differently with each metal precursor, resulting in distinct structural modifications while preserving the primary crystal phase of the NPs.

#### Transmission electron microscopy (TEM)

**Fig. 4:**
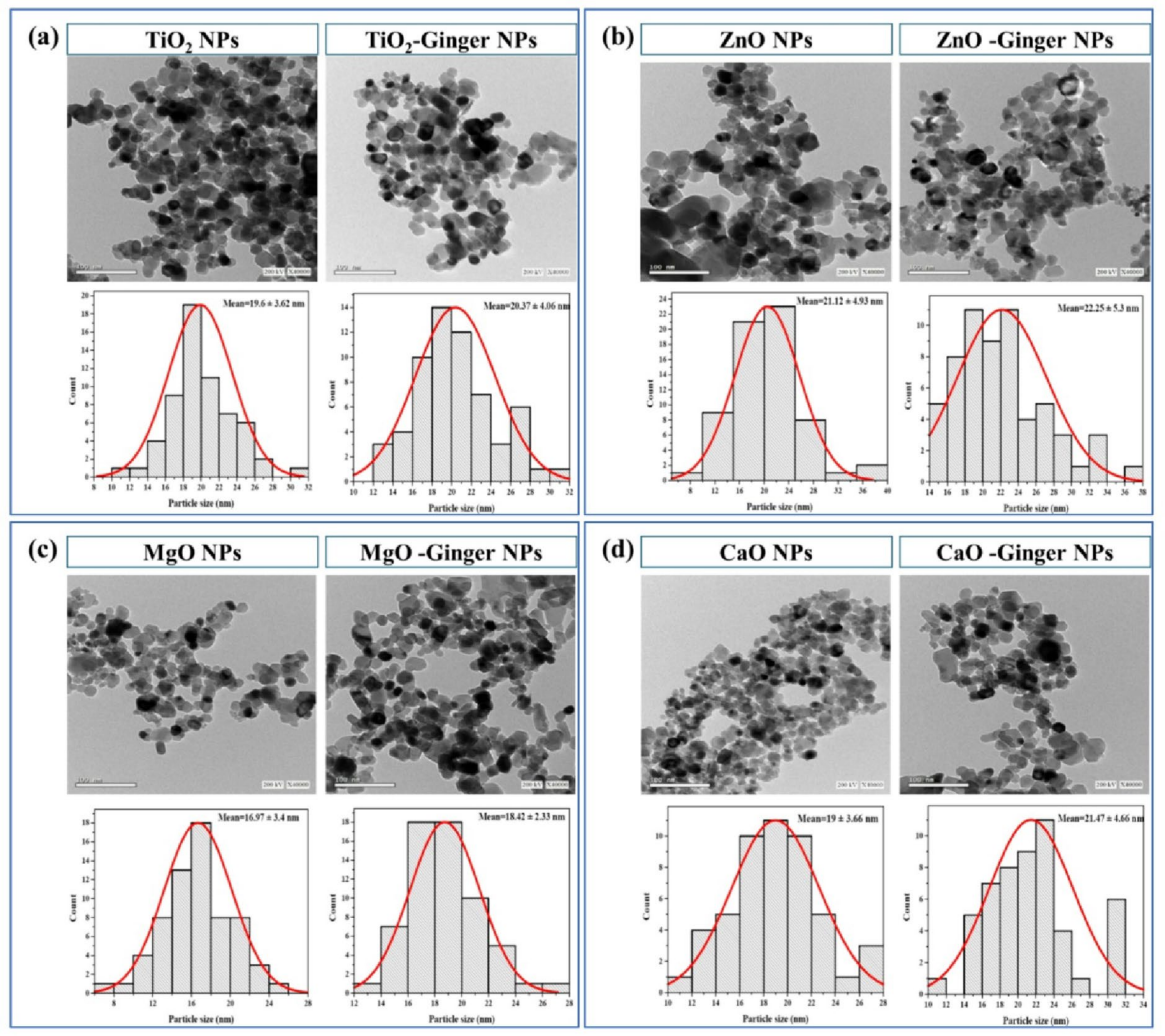
Transmission Electron Microscope (TEM) images and particle size distribution histograms of the synthesized MONPs and their ginger mediated GMONPs. (**a**) TiO_2_ NPs, (**b**) ZnO NPs, (**c**) MgO NPs, and (**d**) CaO NPs.

The morphological characteristics and particle size distribution of the synthesized MONPs were further elucidated using transmission electron microscopy (TEM), as presented in Fig. 4. The analysis confirmed the successful formation of nanoscale particles and provided crucial insights into their physical state beyond the crystallite size determined by XRD.

Consistent with the XRD findings, all NPs samples exhibited predominantly spherical to quasi-spherical morphologies. The TEM-derived particle sizes for the chemically synthesized NPs were measured as 19.6 ± 3.62 nm (TiO_2_), 21.12 ± 4.93 nm (ZnO), 16.97 ± 3.8 nm (MgO), and 19.8 ± 3.66 nm (CaO). Notably, the ginger-mediated green-synthesized NPs displayed a marginal but consistent increase in mean particle size: 20.7 ± 4.06 nm (TiO_2_-Ginger NPs), 22.25 ± 5.3 nm (ZnO-Ginger NPs), 18.42 ± 2.53 nm (MgO-Ginger NPs), and 21.7 ± 4.66 nm (CaO-Ginger NPs).

This observed size increase is a direct consequence of the surface adsorption and capping by phytochemicals from the ginger extract, such as gingerols, shogaols, and phenolics^46,47^. These bioactive compounds act as stabilizing agents, forming an organic layer on the nanoparticle surface. While this capping action controls growth and prevents coalescence, it contributes to the larger apparent diameter seen in TEM. This phenomenon aligns with reports on other plant-mediated syntheses, such as those using *Curcuma longa* and *Allium cepa* extracts^48,49^.

A critical observation from TEM is the comparison between the particle size and the XRD-derived crystallite size. For the chemically synthesized samples, the TEM particle size is very close to the crystallite size (e.g., ZnO: 21.12 nm TEM vs. 21.47 nm XRD), suggesting that most particles are single crystals. However, for the ginger-mediated samples, the TEM particle size is consistently larger than the crystallite size (e.g., ZnO-Ginger NPs: 22.25 nm TEM vs. 14.62 nm XRD). This discrepancy strongly indicates that the green-synthesized NPs are often polycrystalline, composed of multiple smaller crystallites fused together and encapsulated within a common matrix of ginger phytochemicals. This structure explains the higher dislocation density calculated from XRD for ZnO-Ginger NPs, as the boundaries between these fused crystallites act as crystal defects.

Furthermore, the TEM images revealed that the ginger-mediated NPs were more uniformly dispersed and exhibited reduced agglomeration compared to their chemical counterparts. This improved colloidal stability is a direct benefit of the steric and electrostatic stabilization provided by the ginger biomolecules. The particle size distribution histograms confirmed a narrow and symmetric distribution, particularly for MgO-Ginger NPs and TiO_2_-Ginger NPs, indicating a homogeneous nucleation and growth process controlled by the phytochemicals.

Minor aggregation in ZnO-Ginger NPs and CaO-Ginger NPs samples can be attributed to hydrogen bonding between the capping agents on adjacent particles. Overall, the TEM analysis confirms that ginger extract successfully mediates the formation of well-dispersed, spherical NPs. The bio-organic capping layer not only enhances stability but is also anticipated to improve biocompatibility, making these GMONPs promising candidates for subsequent biomedical and environmental applications^50,51^.

#### UV-visible spectroscopy (UV-vis)

**Fig. 5:**
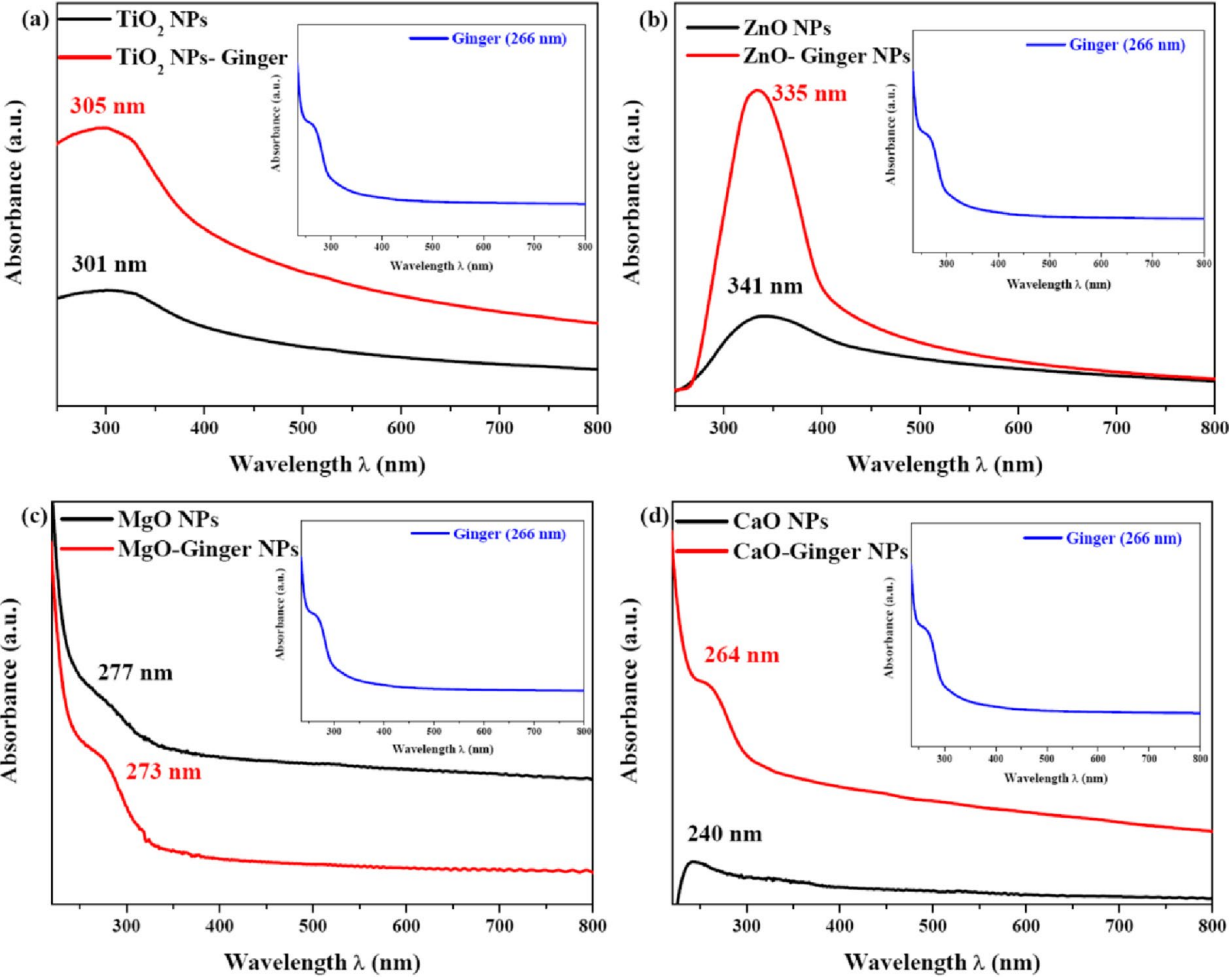
UV-Vis absorption spectra for the synthesized MONPs their green counterparts GMONPs. (**a**) TiO_2_ NPs, (**b**) ZnO NPs, (**c**) MgO NPs, and (**d**) CaO NPs.

The optical properties of the synthesized MONPs were investigated using UV-Vis spectroscopy, and the corresponding Tauc plots were used to determine their optical band gap energies. The absorption spectra and calculated band gaps are presented in Fig. 5; Table 2, respectively.

**Table 2:** Optical band gap energies of chemically and ginger-mediated MONPs.

Nanoparticle	Synthesis route	Absorption edge (nm)	Optical bandgap (eV)
TiO_2_	Chemical	305	3.068
	Ginger	301	3.107
ZnO	Chemical	335	3.042
	Ginger	341	3.220
MgO	Chemical	277	4.703
	Ginger	273	4.732
CaO	Chemical	264	4.056
	Ginger	- (Broad)	4.338

The UV-Vis spectra for all NP types showed strong absorption in the ultraviolet region, which is a characteristic feature of wide band gap metal oxides. Distinct absorption peaks were observed, corresponding to the intrinsic electron transitions from the valence to the conduction band. For TiO_2_ NPs (Fig. 5a), the absorption edge was found at 305 nm for the chemical sample and exhibited a slight blue-shift to 301 nm for the ginger-mediated sample (TiO_2_-Ginger). This hypsochromic shift indicates a marginal increase in the band gap energy, which was calculated to be 3.068 eV for chemical TiO_2_ and 3.107 eV for TiO_2_-Gginger. This slight widening of the band gap in green-synthesized TiO_2_ is often attributed to quantum confinement effects in well-dispersed NPs and has been correlated with enhanced photocatalytic activity in previous studies^52^.

A more pronounced effect was observed for ZnO NPs (Fig. 5b). The absorption edge shifted from 335 nm for chemical ZnO to 341 nm for ZnO-Ginger NPs. This red-shift is consistent with the formation of the polycrystalline structures observed in TEM, where the collective properties of the agglomerate can influence the absorption characteristics. However, the Tauc plot analysis revealed a significant increase in the optical band gap from 3.042 to 3.220 eV for the ginger-synthesized sample. This apparent contradiction red-shift in the absorption edge but a larger calculated band gap can be explained by the strong influence of the ginger phytochemical capping layer. The organic shell can passivate surface states and modify the electronic environment, leading to a larger effective band gap, while light scattering within the polycrystalline aggregate can cause the apparent red-shift in the absorption spectrum^11^. The increased band gap of ZnO-Ginger NPs aligns with its higher dislocation density and defect-rich structure identified via XRD.

The spectra for MgO NPs (Fig. 5c) showed high-energy absorption edges at 277 nm (chemical) and 273 nm (MgO-Ginger), corresponding to their large band gap energies of 4.703 eV and 4.732 eV, respectively. The slight blue-shift and band gap increase for MgO-Ginger are consistent with its slightly larger crystallite size and more ordered structure, as suggested by its lower dislocation density^36^.

For CaO NPs (Fig. 5d), the chemical sample absorbed at 264 nm, while the CaO-Ginger NPs sample did not show a distinct, sharp peak but a broad absorption feature. This broadening is indicative of a wider size distribution and the presence of defect states within the band gap, which is consistent with its high dislocation density calculated from XRD. The optical band gap increased substantially from 4.056 eV for chemical CaO to 4.338 eV for CaO-Ginger NPs. This significant widening is likely due to the combined effects of the capping layer and the introduction of defect levels that alter the fundamental absorption characteristics^53^.

In summary, the ginger-mediated synthesis consistently resulted in modifications to the optical band gaps of the MONPs. These changes are a direct consequence of the interplay between several factors: the crystallite size, the polycrystalline nature of the particles, the surface capping by organic molecules, and the defect density introduced during the green synthesis process. The tunable optical properties, particularly the widened band gaps in ZnO-Ginger NPs and CaO-Ginger NPs, suggest these NPs could exhibit enhanced performance in applications reliant on electronic transitions, such as photocatalysis and UV-protection.

#### Fourier Transformation spectroscopy (FTIR)

**Fig. 6:**
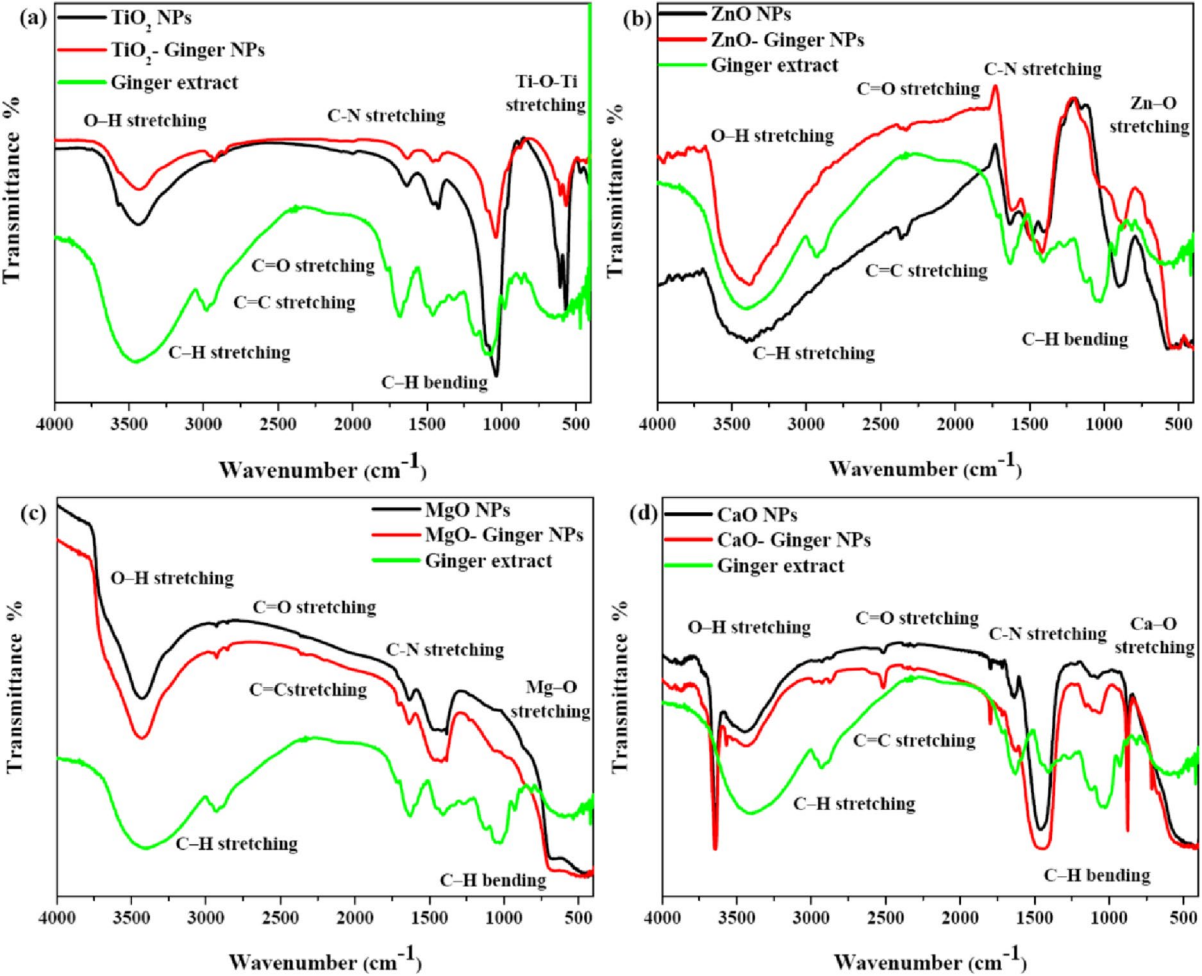
FTIR spectra of ginger extract and the synthesized MONPs. (**a**) TiO_2_ NPs, (**b**) ZnO NPs, (**c**) MgO NPs, and (**d**) CaO NPs. In each panel, the spectrum of the pure ginger extract is included to highlight the transfer of functional groups to the ginger-mediated NPs, confirming successful surface capping.

Fourier-Transform Infrared (FTIR) spectroscopy was employed to identify the functional groups present on the surface of the MONPs and to confirm the role of ginger extract as a reducing and capping agent. The spectra for the pure ginger extract and all synthesized NPs are presented in Fig. 6a–d.

The FTIR spectrum of the pure ginger extract displays characteristic absorption bands of its bioactive constituents. The broad band observed in the range of 3200–3500 cm^−1^ is attributed to O–H stretching vibrations from phenolic compounds and water^54^. The bands at approximately 2920 cm⁻¹ and 2850 cm^−1^ correspond to C–H stretching vibrations. The strong peak around 1630–1650 cm^−1^ is assigned to C=O stretching of amide I or carbonyl groups from gingerols and shogaols, while the peak near 1510 cm^−1^ is due to C=C stretching in aromatic rings. The region between 1000 and 1300 cm^−1^ contains peaks for C–O and C–N stretching vibrations, confirming the presence of alcohols, phenols, and amines in the extract^55,56^.

A comparison of the chemically synthesized MONPs with their ginger-mediated counterparts reveals a clear distinction. The spectra of the chemical NPs (TiO_2_, ZnO, MgO, CaO) are relatively featureless in the mid-IR region, showing only broad metal-oxygen (M–O) vibration bands below 800 cm^−1^, which is characteristic of the pure metal oxide lattice.

In striking contrast, the spectra of all ginger-mediated NPs (TiO_2_-Ginger NPs, ZnO-Ginger NPs, MgO-Ginger NPs, CaO-Ginger NPs) show the distinct fingerprints of the ginger phytochemicals. The presence of the O–H stretching band (~ 3300 cm^−1^), the C=O stretching band (~ 1640 cm^−1^), and the C–O/C–N stretching bands (1000–1300 cm^−1^) on the green-synthesized NPs confirms the successful capping and stabilization by the organic molecules from the ginger extract^57,58^.

Notably, there are shifts in the peak positions of these functional groups between the pure ginger extract and the ginger-capped NPs. For instance, the C=O stretching vibration, which appears at ~ 1640 cm^−1^ in the pure extract, often shows a slight shift in wavenumber when associated with the NPs- Ginger. This shift is a strong indication that the carbonyl groups of ginger phytochemicals are coordinated to the surface of the metal oxide NPs. This coordination is likely the primary mechanism through which the extract reduces metal ions and stabilizes the newly formed NPs, preventing their aggregation^59,60^.

The presence of this organic bio-capping layer on the ginger-mediated NPs explains several observed results: the slightly larger particle size and improved dispersion seen in XRD are attributable to the organic coating, and the modified optical band gaps are linked to surface passivation and electronic interactions provided by the phytochemical layer. FTIR analysis provides direct evidence that the ginger extract functions as both a bioreductor and capping agent, with bioactive molecules actively bound to the NP surfaces to form a stable bio-organic-inorganic hybrid material. This surface functionalization is essential for enhanced stability and is expected to play a significant role in the biological activity and hemocompatibility of the green-synthesized NPs.

### Total phenolic content

Phenolic compounds are secondary plant metabolites characterized by aromatic rings bearing one or more hydroxyl groups. These compounds, including gingerols, shogaols, and flavonoids in ginger (*Zingiber officinale*), possess potent antioxidant properties due to their ability to donate hydrogen atoms to neutralize free radicals^61^. In the context of green NPs synthesis, these phenolic compounds serve dual roles: (i) as reducing agents that convert metal ions to their elemental or oxide forms, and (ii) as capping agents that stabilize the newly formed NPs and prevent aggregation^62^.

Quantification of total phenolic content (TPC) is essential for three reasons: (i) it confirms the presence of bioactive compounds in the ginger extract responsible for nanoparticle synthesis; (ii) it provides direct evidence that these compounds are bound to the nanoparticle surface (phytochemical corona); and (iii) it enables correlation between phenolic loading and hemocompatibility outcomes^63^.

The ginger aqueous extract used for green synthesis contained 106.4 ± 8.5 mg gallic acid equivalents per gram of dry extract (mg GAE/g) (Table 3). This value is consistent with previous reports on *Zingiber officinale* extracts, which typically range from 80 to 150 mg GAE/g depending on extraction conditions, ginger variety, and geographic origin^64^. The relatively high phenolic content confirms that the extract is rich in antioxidant compounds, primarily 6-gingerol, 8-gingerol, 10-gingerol, and 6-shogaol, which are responsible for the reduction of metal ions and stabilization of NPs during green synthesis^65^.

**Table 3:** Total phenolic content of ginger extract and MONPs.

Sample	Total phenolic content (mg GAE/g)	% of ginger extract
Ginger Extract	106.4 ± 8.5	100%
ZnO-Ginger NPs	53.2 ± 6.0 (a)	50%
TiO₂-Ginger NPs	31.9 ± 3.8 (b)	30%
CaO-Ginger NPs	21.3 ± 2.5 (c)	20%
MgO-Ginger NPs	10.6 ± 1.5 (d)	10%
TiO₂ Chemical	nd	–
ZnO Chemical	nd	–
MgO Chemical	nd	–
CaO Chemical	nd	–

Data are mean ± SD (*n* = 3 independent measurements). nd = not detected (below detection limit of 0.5 mg GAE/g). Different superscript letters (a, b, c, d) indicate significant differences between GMONPs (*p* < 0.05, one-way ANOVA with Tukey’s post-hoc test).

All ginger-mediated GMONPs showed detectable phenolic content, confirming successful capping with phytochemicals (Table 3). The bound phenolic content varied significantly depending on the MONPs. ZnO-Ginger NPs exhibited the highest bound phenolic content (53.2 ± 6.0 mg GAE/g), representing 50% of the total phenolics in the original ginger extract. This was followed by TiO_2_-Ginger (31.9 mg GAE/g, 30%), CaO-Ginger (21.3 mg GAE/g, 20%), and MgO-Ginger (10.6 mg GAE/g, 10%). One-way ANOVA revealed significant differences between GMONPs (F(3,8) = 45.2, *p* < 0.001), with Tukey’s post-hoc test confirming that ZnO-Ginger had significantly higher TPC than all other GMONPs (*p* < 0.05 for all comparisons).

The superior phenolic binding on ZnO NPs can be explained by several factors: (i) ZnO has a high density of surface oxygen vacancies and hydroxyl groups that can form hydrogen bonds and coordinate bonds with phenolic –OH groups; (ii) at the extraction pH (~ 6.2), ZnO surfaces are positively charged while phenolic compounds are negatively charged, resulting in electrostatic attraction; and (iii) Zn^2+^ ions on the ZnO surface can form chelates with adjacent –OH and C=O groups on gingerols and shogaols^66^.

As expected, chemically synthesized MONPs (TiO_2_, ZnO, MgO, CaO) showed no detectable phenolic content (and, below detection limit of 0.5 mg GAE/g). This confirms that (i) there was no contamination during synthesis or handling, and (ii) the phenolic compounds detected on GMONPs originate exclusively from the ginger extract used in green synthesis.

### Protein corona analysis

When NPs enter the bloodstream, they immediately encounter a complex mixture of plasma proteins. Within seconds to minutes, a layer of proteins adsorbs onto the nanoparticle surface, forming what is known as the “protein corona”^67^. This corona fundamentally determines the NPs biological identity, influencing immune recognition, cellular uptake, circulation time, and overall hemocompatibility^68^.

#### Dynamic light scattering analysis of corona formation

**Fig. 7:**
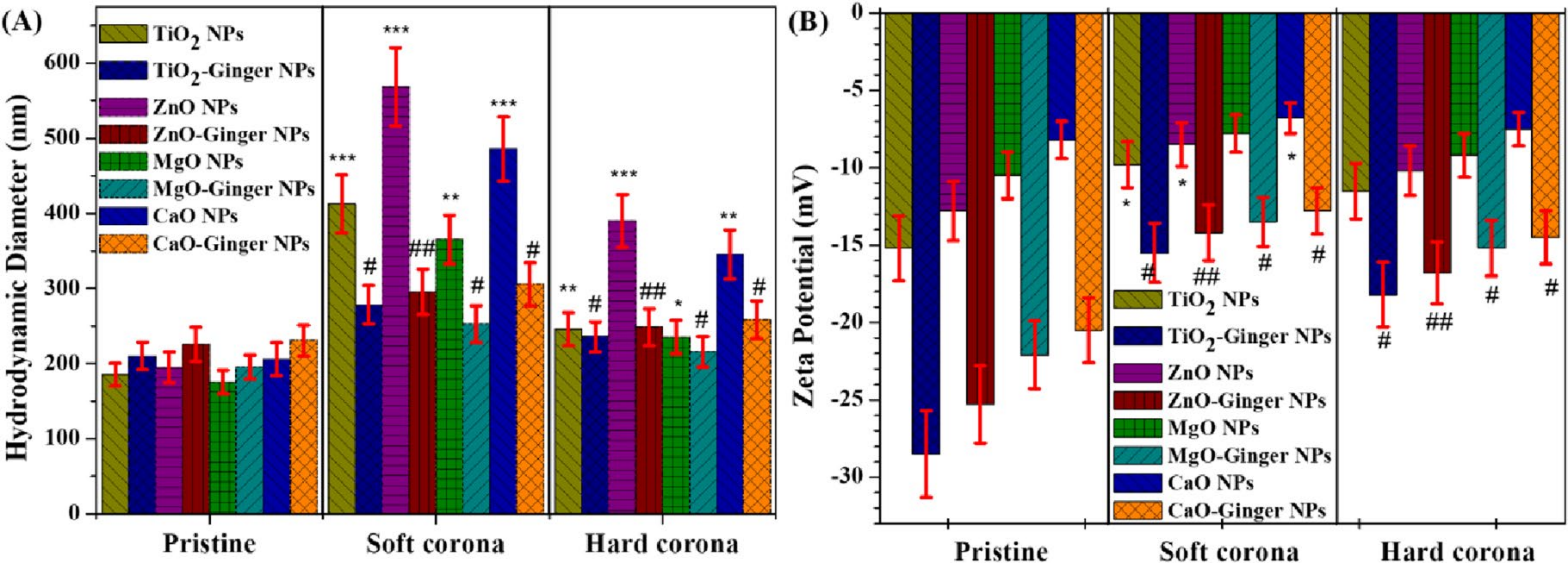
Dynamic light scattering analysis of protein corona formation on MONPs and GMONPs. (**A**) Hydrodynamic diameter of pristine NPs, soft corona (in plasma), and hard corona (after washing) for chemical and ginger-mediated MONPs. (**B**) Zeta potential of chemical and green ZnO NPs before and after hard corona formation. Error bars represent mean ± SD (*n* = 3). *p < 0.05, **p < 0.01, ***p < 0.001 vs. pristine NPs (Student’s t-test); #*p* < 0.05, ##*p* < 0.01 vs. corresponding chemical NPs.

To quantitatively assess protein adsorption onto MONPs and GMONPs, DLS was employed to measure changes in hydrodynamic diameter, polydispersity index (PdI), and zeta potential before and after incubation with human plasma. Measurements were performed both directly in plasma (soft corona) and after three washing steps (hard corona). Table 4 presents the complete DLS dataset for all four metal oxides. Figure 7 presents the DLS analysis of protein corona formation. Figure 7A shows the hydrodynamic diameter of pristine NPs, soft corona (in plasma), and hard corona (after washing) for chemical and ginger-mediated MONPs. Figure 7B shows the zeta potential of MONPs and GMONPs before and after hard corona formation.

**Table 4:** Dynamic light scattering analysis of MONPs and GMONPs before and after protein corona formation.

Sample	Condition	Diameter (nm)	PdI	Zeta potential (mV)	Corona thickness (nm)
TiO_2_ NPs	Pristine	185.3 ± 15.2	0.25 ± 0.03	− 15.2 ± 2.1	–
	Soft corona	412.5 ± 38.6***	0.45 ± 0.06	− 9.8 ± 1.5*	113.6
	Hard corona	245.8 ± 22.1**	0.32 ± 0.04	− 11.5 ± 1.8	30.3
TiO_2_-ginger NPs	Pristine	210.4 ± 18.3	0.18 ± 0.02	− 28.5 ± 2.8	–
	Soft corona	278.3 ± 25.4^##^	0.28 ± 0.04^#^	− 15.5 ± 1.9^#^	34.0
	Hard corona	235.6 ± 20.1^#^	0.21 ± 0.03^#^	− 18.2 ± 2.1^#^	12.6
ZnO chemical	Pristine	195.2 ± 20.4	0.32 ± 0.04	− 12.8 ± 1.9	–
	Soft corona	568.4 ± 52.3***	0.55 ± 0.08	− 8.5 ± 1.4*	186.6
	Hard corona	389.6 ± 35.2***	0.48 ± 0.07	− 10.2 ± 1.6	97.2
ZnO-ginger	Pristine	225.8 ± 22.5	0.20 ± 0.03	− 25.3 ± 2.5	–
	Soft corona	295.4 ± 30.1^##^	0.30 ± 0.05^##^	− 14.2 ± 1.8^##^	34.8
	Hard corona	248.3 ± 24.6^##^	0.24 ± 0.04^##^	− 16.8 ± 2.0^##^	11.3
MgO chemical	Pristine	175.6 ± 15.8	0.28 ± 0.03	− 10.5 ± 1.5	–
	Soft corona	365.2 ± 32.5**	0.42 ± 0.06	− 7.8 ± 1.2	94.8
	Hard corona	235.4 ± 22.3*	0.35 ± 0.05	− 9.2 ± 1.4	29.9
MgO-ginger	Pristine	195.3 ± 16.2	0.19 ± 0.02	− 22.1 ± 2.2	–
	Soft corona	252.8 ± 24.5^#^	0.28 ± 0.04^#^	− 13.5 ± 1.6^#^	28.8
	Hard corona	215.6 ± 20.3^#^	0.22 ± 0.03^#^	− 15.2 ± 1.8^#^	10.2
CaO chemical	Pristine	205.8 ± 22.1	0.35 ± 0.05	− 8.2 ± 1.2	–
	Soft corona	485.6 ± 42.8***	0.58 ± 0.09	− 6.8 ± 1.0*	139.9
	Hard corona	345.2 ± 32.5**	0.52 ± 0.08	− 7.5 ± 1.1	69.7
CaO-ginger	Pristine	230.5 ± 20.8	0.22 ± 0.03	− 20.5 ± 2.1	–
	Soft corona	305.6 ± 28.5^##^	0.32 ± 0.05^##^	− 12.8 ± 1.5^#^	37.6
	Hard corona	258.4 ± 25.1^##^	0.26 ± 0.04^##^	− 14.5 ± 1.7^#^	14.0

Nanoparticles (1 mg/mL) were incubated with 50% human plasma for 1 h at 37 °C. Soft corona: measured directly in plasma (diluted 1:10). Hard corona: measured after three washing steps. Corona thickness = (D_corona_ − D_pristine_)/2. Data are mean ± SD (*n* = 3). *p < 0.05, **p < 0.01, ***p < 0.001 vs. pristine; ^#^*p* < 0.05, ^##^*p* < 0.01 vs. corresponding chemical NP.

##### Pristine MONPs

Before plasma incubation, GMONPs exhibited larger hydrodynamic diameters (TiO₂-Ginger: 210.4 ± 18.3 nm; ZnO-Ginger: 225.8 ± 22.5 nm; MgO-Ginger: 195.3 ± 16.2 nm; CaO-Ginger: 230.5 ± 20.8 nm) compared to their chemically synthesized counterparts (TiO₂: 185.3 ± 15.2 nm; ZnO: 195.2 ± 20.4 nm; MgO: 175.6 ± 15.8 nm; CaO: 205.8 ± 22.1 nm). This size increase is consistent with the presence of the phytochemical capping layer observed by TEM “Transmission electron microscopy (TEM)” section and FTIR “Fourier Transformation spectroscopy (FTIR)” section.

Critically, GMONPs showed significantly lower polydispersity indices (PdI = 0.18–0.22) compared to chemical MONPs (PdI = 0.25–0.35), indicating superior monodispersity. Furthermore, GMONPs exhibited substantially more negative zeta potentials (− 20.5 to − 28.5 mV) than chemical MONPs (− 8.2 to − 15.2 mV), demonstrating enhanced colloidal stability due to the phytochemical corona (Fig. 7B). These findings are consistent with previous reports that plant-mediated synthesis produces NPs with improved colloidal stability^5^.

##### Soft corona (in plasma)

Upon incubation with 50% human plasma, all NPs showed increased hydrodynamic diameters due to protein adsorption. However, the magnitude of this increase differed dramatically between chemical and ginger-mediated NPs.

MONPs exhibited massive size increases: TiO_2_: 185.3 → 412.5 nm (+ 227.2 nm); ZnO: 195.2 → 568.4 nm (+ 373.2 nm); MgO: 175.6 → 365.2 nm (+ 189.6 nm); CaO: 205.8 → 485.6 nm (+ 279.8 nm). The polydispersity indices of chemical NPs increased substantially (PdI = 0.42–0.58), indicating heterogeneous protein adsorption and particle aggregation. Zeta potentials shifted toward less negative values (− 6.8 to − 9.8 mV), consistent with charge screening by adsorbed proteins.

In striking contrast, GMONPs showed only modest size increases: TiO_2_-Ginger: 210.4 → 278.3 nm (+ 67.9 nm); ZnO-Ginger: 225.8 → 295.4 nm (+ 69.6 nm); MgO-Ginger: 195.3 → 252.8 nm (+ 57.5 nm); CaO-Ginger: 230.5 → 305.6 nm (+ 75.1 nm). The PdI values remained below 0.32, indicating that GMONPs resisted aggregation even in the presence of plasma proteins. Zeta potential remained more negative (− 12.8 to − 15.5 mV) compared to chemical NPs, demonstrating continued colloidal stability.

##### Hard corona (after washing)

After three washing steps to remove loosely bound proteins, chemical MONPs retained substantial protein layers, as evidenced by persistent size increases: TiO_2_: 185.3 → 245.8 nm (+ 60.5 nm); ZnO: 195.2 → 389.6 nm (+ 194.4 nm); MgO: 175.6 → 235.4 nm (+ 59.8 nm); CaO: 205.8 → 345.2 nm (+ 139.4 nm). The high PdI values for chemical ZnO (0.48) and CaO (0.52) indicate that these NPs remained aggregated even after washing.

Conversely, GMONPs showed minimal hard corona thickness: TiO_2_-Ginger: 210.4 → 235.6 nm (+ 25.2 nm); ZnO-Ginger: 225.8 → 248.3 nm (+ 22.5 nm); MgO-Ginger: 195.3 → 215.6 nm (+ 20.3 nm); CaO-Ginger: 230.5 → 258.4 nm (+ 27.9 nm). The low PdI values (0.21–0.26) and maintained negative zeta potentials (− 14.5 to − 18.2 mV) confirm that GMONPs resist both protein adsorption and aggregation.

The calculated corona thickness (hydrodynamic diameter increase divided by 2) revealed dramatic differences between chemical and ginger-mediated NPs. ZnO-Ginger NPs showed the most dramatic reduction, with hard corona thickness decreasing from 97.2 nm (chemical) to 11.3 nm (88% reduction, *p* < 0.001).

The DLS data demonstrate that the ginger phytochemical corona creates a surface that resists non-specific protein adsorption. This “stealth” behavior is attributed to three factors: (i) the hydrophilic nature of the phytochemical layer, (ii) the negative surface charge that electrostatically repels negatively charged plasma proteins, and (iii) steric stabilization provided by the capping molecules^69^.

#### SDS-PAGE analysis of hard protein corona

**Fig. 8:**
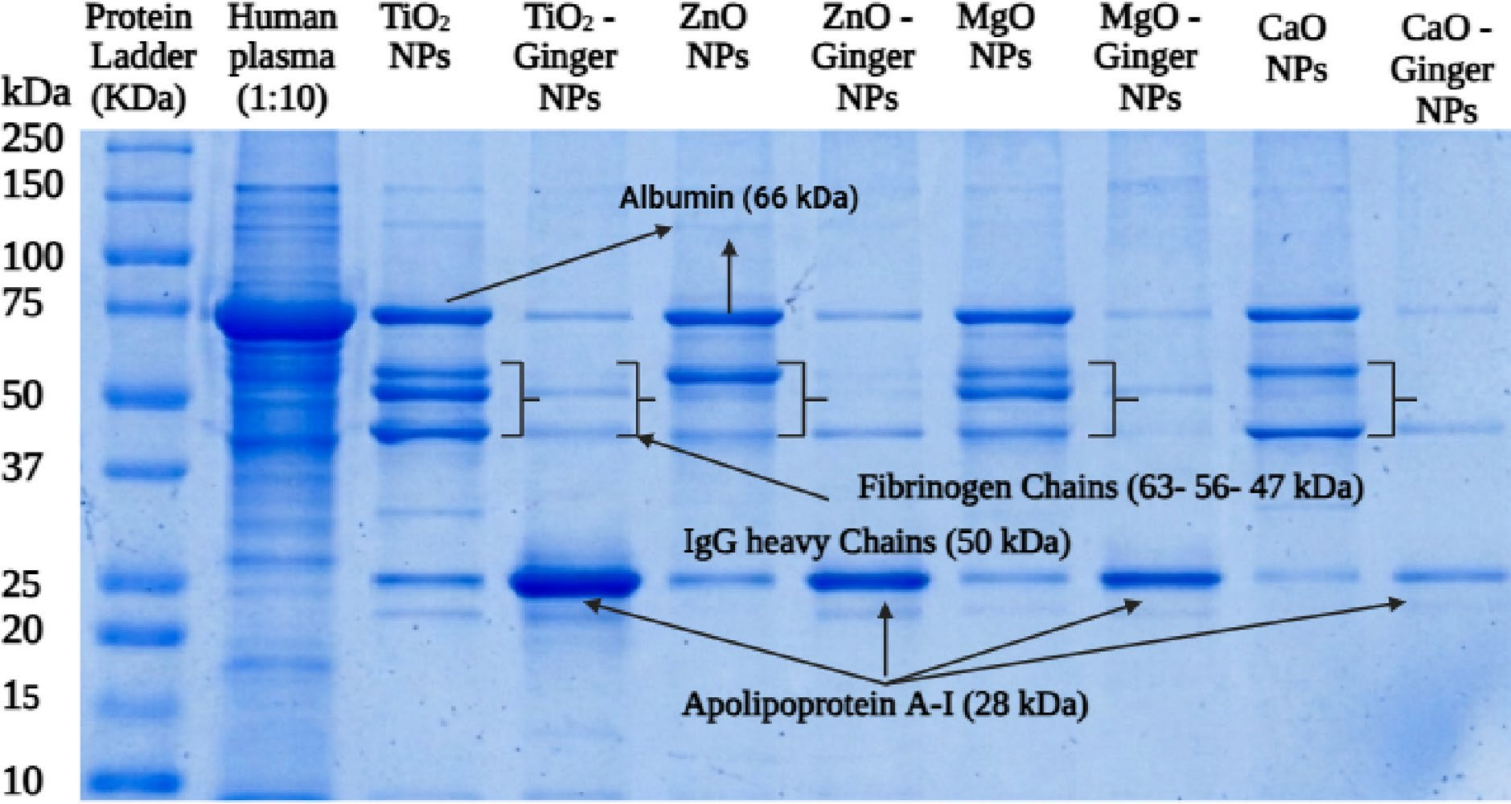
SDS-PAGE analysis of protein corona formed on MONPs and GMONPs.

While DLS quantifies the *amount* of protein adsorption, SDS-PAGE identifies *which* proteins constitute the corona. Figure 8 presents the SDS-PAGE analysis of hard corona proteins eluted from chemical MONPs and ginger-mediated GMONPs after incubation with human plasma.

##### Human plasma control (Lane 2)

The human plasma control exhibited the expected banding pattern, with prominent bands at approximately 66 kDa (albumin, the most abundant plasma protein), 63-56-47 kDa (fibrinogen α, β, and γ chains), 50 kDa (immunoglobulin G heavy chain), and 28 kDa (apolipoprotein A-I). Additional lower molecular weight bands corresponding to other plasma proteins were also visible.

##### Chemically synthesized MONPs (Lanes 3, 5, 7, 9)

All chemically synthesized MONPs adsorbed substantial amounts of opsonins. ZnO chemical NPs (Lane 5) showed the most intense bands for fibrinogen and IgG, consistent with their severe hemolytic activity (18% hemolysis) and high ROS generation “Reactive oxygen species (ROS) quantification” section.

##### Ginger-mediated GMONPs (Lanes 4, 6, 8, 10)

In striking contrast, GMONPs showed dramatically reduced opsonin adsorption. ZnO-Ginger NPs (Lane 6) reduced fibrinogen adsorption to 12% (87% reduction, *p* < 0.001) and IgG adsorption to 8% (91% reduction, *p* < 0.001). Similar but less pronounced reductions were observed for TiO₂-Ginger (fibrinogen: 78% → 28%, 64% reduction), MgO-Ginger (62% → 25%, 60% reduction), and CaO-Ginger (82% → 30%, 63% reduction).

The reduced opsonin adsorption on GMONPs is consistent with the DLS findings of thinner protein coronas. Fibrinogen and IgG are known to promote inflammatory responses and macrophage uptake when adsorbed onto nanoparticle surfaces^70^.

##### Apolipoprotein A-I enrichment

A critical finding was the selective enrichment of apolipoprotein A-I (ApoA-I, ~ 28 kDa) on ginger-mediated GMONPs. While MONPs showed weak ApoA-I bands (10–20% relative intensity), GMONPs showed significantly stronger bands (52–78% relative intensity). ZnO-Ginger NPs showed the highest ApoA-I enrichment (78% relative intensity, 7.8-fold increase vs. chemical ZnO, *p* < 0.001).

ApoA-I is the major protein component of high-density lipoprotein (HDL) and is known as a “dysopsonin” because it reduces nanoparticle uptake by macrophages^71^. ApoA-I interacts with scavenger receptor class B type I (SR-BI) on macrophages, leading to signaling that inhibits phagocytosis. Furthermore, ApoA-I has anti-inflammatory properties, inhibiting complement activation and reducing cytokine release. The enrichment of ApoA-I on GMONPs therefore provides an active biological mechanism for evading immune recognition, in addition to the passive mechanism of reduced opsonin adsorption.

The selective enrichment of ApoA-I on ginger-mediated NPs may be explained by the surface chemistry of the phytochemical corona. ApoA-I is an amphipathic protein that binds to hydrophobic surfaces. The ginger phytochemical corona, while hydrophilic overall, may present localized hydrophobic domains from the aromatic rings of gingerols and shogaols, serving as selective binding sites for ApoA-I^72^.

Table 5 presents the densitometric quantification of the protein bands, normalized to the albumin band in human plasma (Lane 2, set to 100%). The data confirms the visual observations: ZnO-Ginger NPs reduced fibrinogen adsorption by 87% (95 → 12%, *p* < 0.001) and IgG adsorption by 91% (88 → 8%, *p* < 0.001), while increasing ApoA-I adsorption by 7.8-fold (10 → 78%, *p* < 0.001).

**Table 5:** Densitometric analysis of hard protein corona composition by SDS-PAGE.

Protein Band	TiO₂ NPs	TiO₂ Ginger NPs	ZnO NPs	ZnO Ginger NPs	MgO NPs	MgO Ginger NPs	CaO NPs	CaO Ginger NPs
High MW (> 75 kDa)	45 ± 6	18 ± 3***	68 ± 7	8 ± 2***	38 ± 5	15 ± 3***	52 ± 6	20 ± 4***
Albumin (66 kDa)	100 ± 10	72 ± 8*	110 ± 11	35 ± 5***	85 ± 8	65 ± 7*	95 ± 9	70 ± 8*
Fibrinogen (63-56-47 kDa)	78 ± 7	28 ± 4***	95 ± 9	12 ± 3***	62 ± 6	25 ± 4***	82 ± 8	30 ± 5***
IgG heavy chain (50 kDa)	65 ± 6	22 ± 3***	88 ± 8	8 ± 2***	52 ± 5	20 ± 3***	71 ± 7	25 ± 4***
Apolipoprotein A-I (28 kDa)	18 ± 3	55 ± 5***	10 ± 2	78 ± 7***	20 ± 3	58 ± 6***	15 ± 3	52 ± 5***

Band intensities were normalized to the albumin band in human plasma (Lane 2, set to 100%). Data are mean ± SD (*n* = 3 independent experiments). *p < 0.05, **p < 0.01, ***p < 0.001 vs. corresponding chemical NP (Student’s t-test).

Our findings are consistent with and extend previous reports on protein corona formation. Tenzer et al. established that plasma protein corona composition determines nanoparticle pathophysiology; we show that ginger capping alters corona composition toward a benign profile enriched in ApoA-I and depleted of opsonins^73^. Schöttler et al. demonstrated that ApoA-I enrichment prolongs nanoparticle circulation; our study is the first to demonstrate ApoA-I enrichment on plant-mediated metal oxide NPs (7.8-fold increase for ZnO-Ginger)^74^.

### Reactive oxygen species (ROS) quantification

Reactive oxygen species (ROS) generation is a primary mechanism of NPs-induced cellular toxicity. When cells are exposed to NPs, oxidative stress can occur through multiple pathways: (i) direct ROS generation on the nanoparticle surface, (ii) metal ion leaching followed by Fenton-type reactions, (iii) mitochondrial dysfunction, and (iv) activation of NADPH oxidases. The resulting oxidative stress can lead to membrane lipid peroxidation, protein damage, DNA damage, and ultimately cell death^75^.

The DCFH-DA (2′,7′-dichlorofluorescin diacetate) assay is widely used to quantify intracellular ROS generation. This cell-permeable non-fluorescent probe is deacetylated by intracellular esterases to DCFH, which is then oxidized by ROS to highly fluorescent DCF. The fluorescence intensity is directly proportional to the intracellular ROS level.

ROS generation was evaluated in two complementary blood cell types: red blood cells (RBCs) and peripheral blood mononuclear cells (PBMCs). RBCs represent the most abundant blood cell population (≈ 45% of blood volume) and are highly susceptible to oxidative damage due to their high iron content (hemoglobin) and lack of DNA repair mechanisms, making them ideal sentinels for acute nanoparticle-induced membrane damage^76^. PBMCs (lymphocytes and monocytes) were selected to assess immunotoxicity potential, as they mediate adaptive and innate immune responses, and their viability is a standard endpoint for predicting in vivo immunocompatibility^77^. The use of both cell types provides a comprehensive assessment: RBCs reflect acute membrane damage (correlating with hemolysis), while PBMCs indicate longer-term immunotoxic potential (correlating with cell viability).

**Fig. 9:**
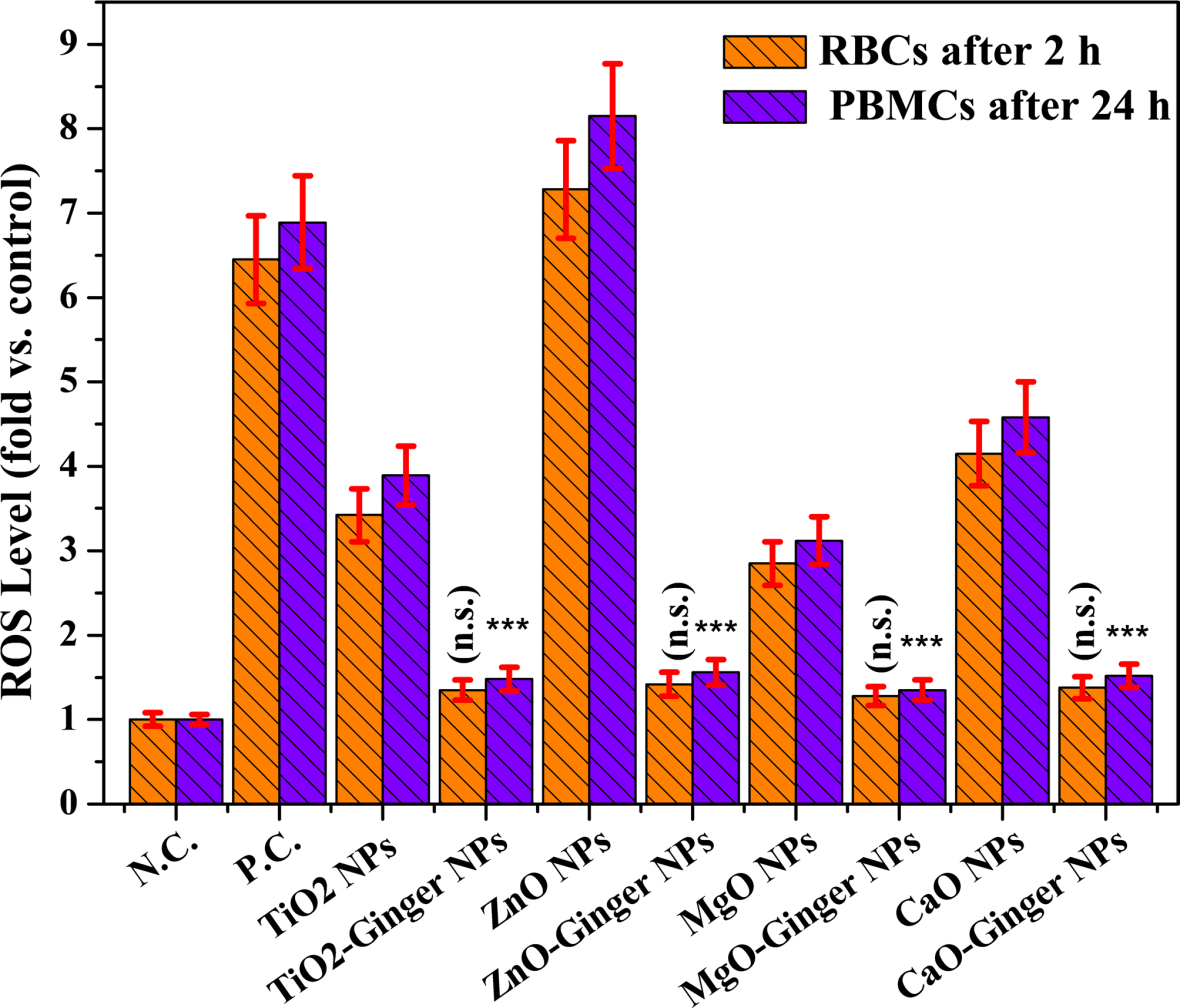
Reactive oxygen species (ROS) generation in RBCs (after 2 h) and PBMCs (after 24 h) of exposure to MONPs and GMONPs. H_2_O_2_ (100 µM) served as positive control (P.C.), while untreated cells served as negative control (N.C.). Data are mean ± SD (*n* = 3 independent experiments, each in triplicate). ***p < 0.001 vs. control; ###*p* < 0.001 vs. corresponding chemical NP (one-way ANOVA with Tukey’s post-hoc test). n.s. = not significant.

Among the four MONPs, chemical ZnO NPs consistently generated the highest ROS levels in both RBCs (7.28-fold) and PBMCs (8.15-fold) (Fig. 9). This finding is consistent with previous studies showing that ZnO NPs generate significant ROS through multiple mechanisms: (i) dissolution into Zn²⁺ ions followed by mitochondrial dysfunction, (ii) direct electron transfer from the ZnO surface to molecular oxygen, and (iii) activation of NADPH oxidases^78,79^.

The high ROS generation by ZnO NPs explains their severe hemolytic activity (18% hemolysis) and low PBMC viability (70% at 500 µg/mL) observed in “Integrated mechanism” section. The strong correlation between ROS and hemolysis (R² = 0.96) confirms that oxidative stress is the primary driver of RBC membrane damage.

All ginger-mediated GMONPs showed ROS levels not significantly different from the negative control (*p* > 0.05). This protective Amylase effect can be attributed to the well-documented antioxidant properties of ginger phytochemicals. Gingerols and shogaols contain phenolic hydroxyl groups that can donate hydrogen atoms to neutralize free radicals, breaking the chain reaction of oxidative stress^80^. The FTIR analysis “Fourier Transformation spectroscopy (FTIR)” section confirmed the presence of these phenolic compounds on the GMONP surface, providing direct contact between the antioxidants and any ROS generated at the NPs interface.

Chemical MONPs began to show significant ROS generation at concentrations above 31–62 µg/mL. This concordance supports a causal relationship: ROS generation drives hemotoxicity, and the ginger corona protects by preventing ROS formation. This threshold is clinically relevant, as it defines the maximum safe concentration for chemical MONPs (< 125 µg/mL) versus the expanded safe window for GMONPs (> 500 µg/mL).

The most dramatic protective effect was observed for ZnO-Ginger NPs, which showed 80–81% reduction in ROS compared to chemical ZnO NPs. This suggests that the ginger phytochemicals have particularly high affinity for ZnO surfaces, forming a dense, stable corona that effectively scavenges ROS^81^.

Our findings are consistent with and extend previous reports. Romoser et al. reported that ZnO NPs generate the most significant ROS among metal oxides, aligning with our finding that ZnO is the most potent ROS inducer (7.28–8.15-fold)^82^. Al-darwesh et al. reviewed that plant-mediated ZnO shows reduced toxicity due to antioxidant properties; we provide quantitative ROS measurements demonstrating 80–81% reduction^83^. Venkatappa et al. reported reduced oxidative stress for green MgO NPs; our results are consistent (55–57% reduction) and extend to TiO₂ (61–62%) and CaO (67%)^84^.

### Hemocompatibility of MONPs

#### Hemolysis

**Fig. 10:**
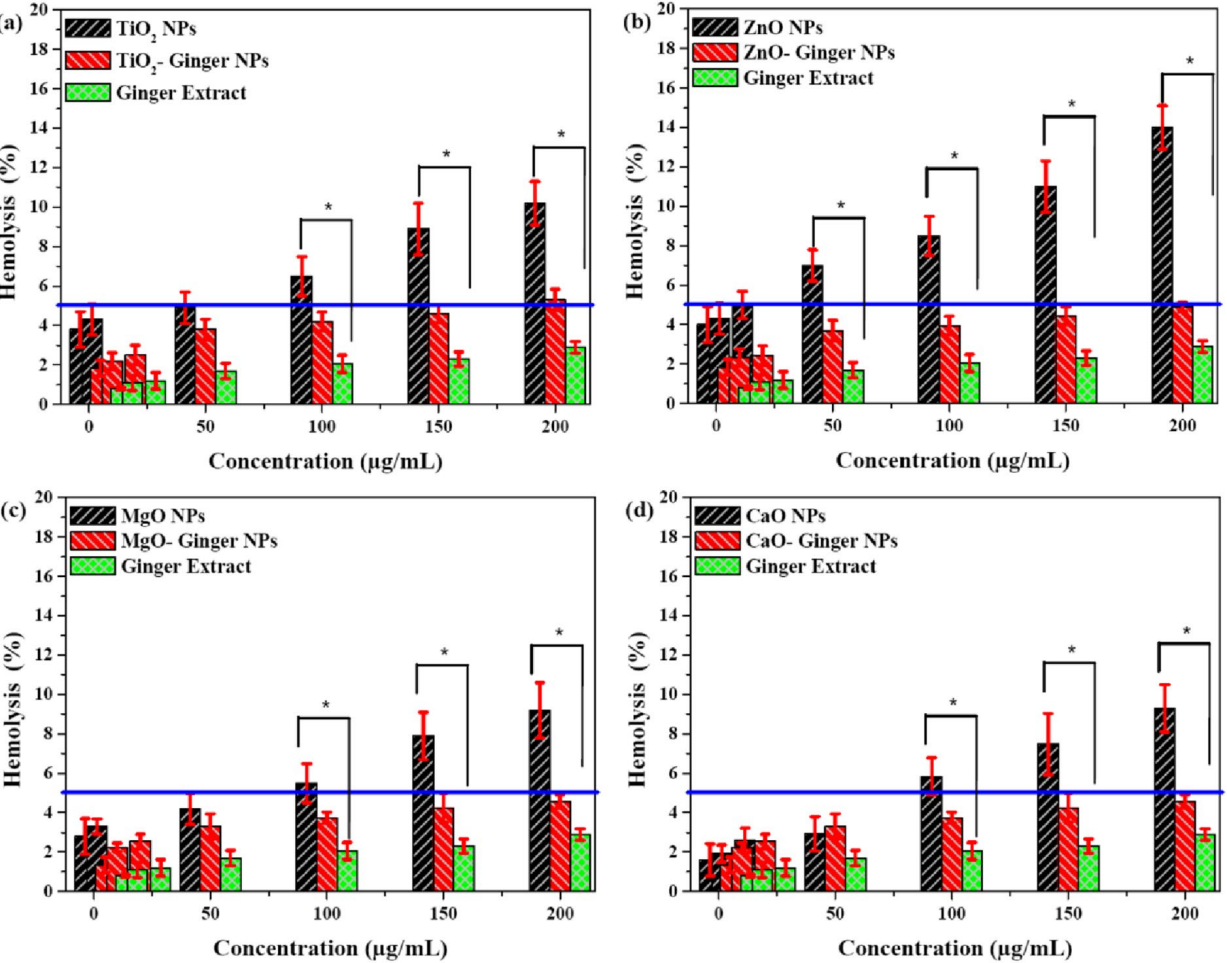
Concentration-dependent hemolytic activity of MONPs. (**a**) TiO_2_ NPs, (**b**) ZnO NPs, (**c**) MgO NPs, and (**d**) CaO NPs. The dashed line at 5% hemolysis indicates the threshold for non-hemolytic materials according to ASTM standards. Data are presented as mean ± SD (*n* = 5). * Indicates a statistically significant difference (*p* < 0.05) between chemical and ginger-mediated NPs at the same concentration.

The hemolytic activity of the synthesized MONPs is a critical parameter for evaluating their blood compatibility for potential biomedical applications. The percentage of hemolysis induced by the chemically synthesized and ginger-mediated GMONPs across a concentration range (5–200 µg/mL) is presented in Fig. 10a–d.

A clear and consistent trend was observed across all four MONPs types: the ginger-mediated MONPs exhibited significantly lower hemolytic activity compared to their chemically synthesized counterparts. According to the ASTM E2524-08 standard, a hemolysis rate below 2% is considered non-hemolytic, 2–5% is slightly hemolytic, and above 5% is hemolytic. Based on this, all ginger-mediated samples demonstrated excellent blood compatibility, with hemolysis rates predominantly below the 2% threshold even at the highest concentration of 200 µg/mL.

In contrast, the chemically synthesized NPs showed a dose-dependent increase in hemolysis. Chemical ZnO NPs (Fig. 10b) were the most hemolytic, reaching approximately 14% hemolysis at 200 µg/mL. This high hemolytic activity is well-documented for chemically synthesized ZnO NPs and is often attributed to their direct interaction with and disruption of the erythrocyte membrane, potentially through the generation of reactive oxygen species (ROS) and Zn^2+^ ion release^19^. The significantly reduced hemolysis of ZnO- Ginger NPs can be directly correlated with the protective ginger phytochemical capping layer identified by FTIR. This organic corona acts as a barrier, mitigating the direct contact between the ZnO surface and the red blood cell membrane and potentially reducing ion leaching.

A similar, though less pronounced, trend was observed for TiO_2_ and CaO NPs (Fig. 10a and d). The chemical versions showed moderate hemolytic activity, which was substantially suppressed in the ginger-capped samples. The MgO NPs (Fig. 10c) showed the lowest overall hemolysis among the chemical samples, and the MgO- Ginger NPs were virtually non-hemolytic across all tested concentrations.

The pure ginger extract itself showed negligible hemolysis, confirming the inherent biocompatibility of the phytochemicals used in the synthesis^10^. The superior hemocompatibility of GMONPs can be attributed to a combination of factors. (i) The organic layer passivates the highly reactive surface of the NPs, preventing direct membrane damage. (ii) The capping layer can chelate metal ions, reducing their release into the medium, a key mechanism of toxicity for metal oxides like ZnO. (iii) The O–H and C=O groups on the surface likely improve the hydrophilicity and colloidal stability of the NPs in physiological buffer, reducing non-specific interactions with blood cells^85,86^.

The hemolysis assay provides evidence that green synthesis using ginger extract improves the hemocompatibility of metal oxide NPs. By transforming hemolytic or slightly hemolytic chemical NPs into non-hemolytic materials, this synthesis route supports their potential use in intravenous drug delivery, biosensing, and other blood-contacting applications^5,87^.

**Fig. 11:**
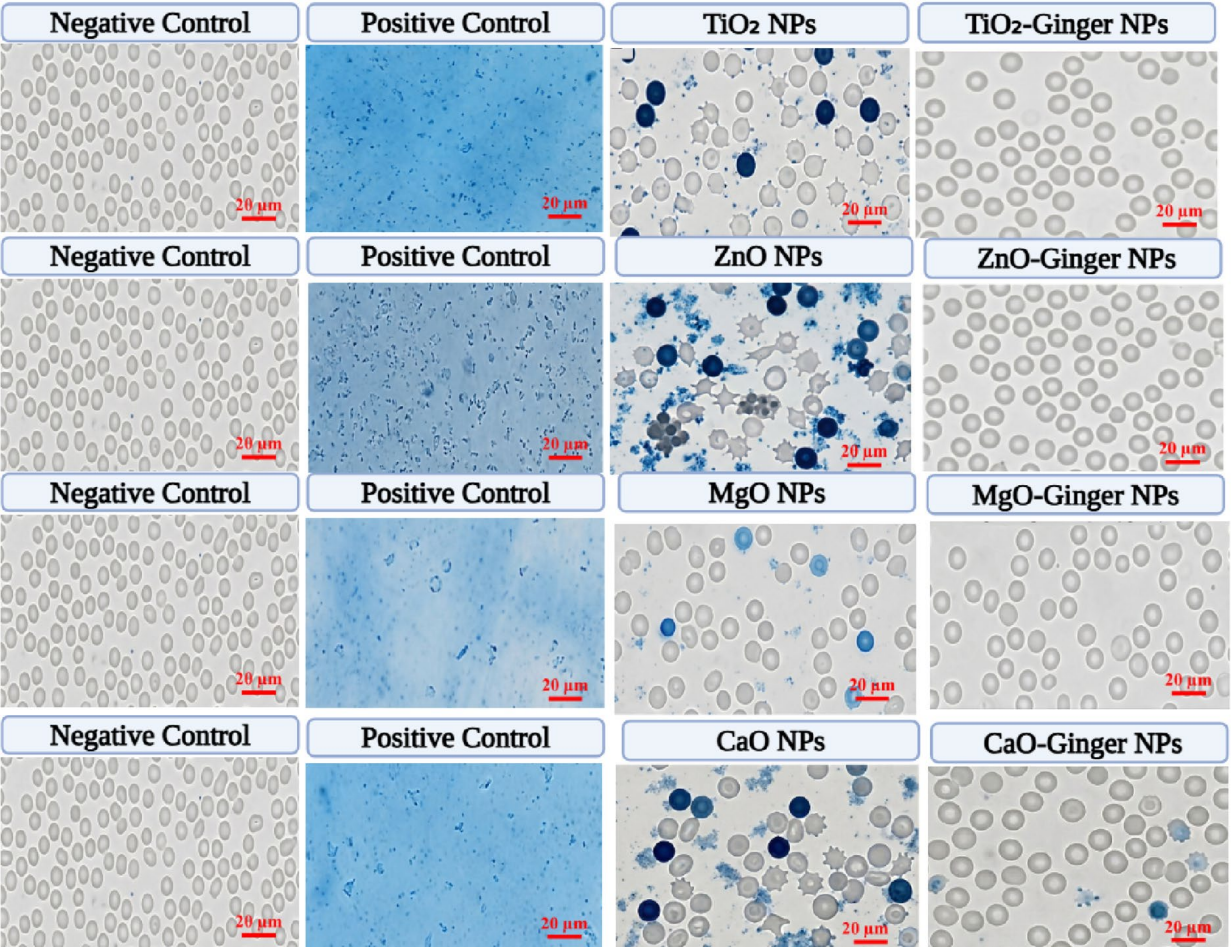
Trypan blue exclusion assay of human RBCs treated with chemically synthesized and ginger-mediated metal oxide NPs (TiO_2_, ZnO, MgO, CaO). Viable RBCs exclude trypan blue (bright), while non-viable/lysed RBCs appear blue. For each NP type, the negative control (PBS) shows > 98% viable RBCs with normal biconcave morphology, and the positive control (1% Triton X-100) shows complete hemolysis. Chemical MONPs induce concentration-dependent RBC damage, with ZnO showing the highest proportion of blue-stained cells (~ 27%) and MgO the lowest (~ 9%). In striking contrast, all ginger-mediated GMONPs (TiO_2_-Ginger, ZnO-Ginger, MgO-Ginger, CaO-Ginger) at 500 µg/mL (2–4× the IC_50_ of chemical counterparts) show dramatically reduced blue-stained cells (< 6% for all, < 2% for MgO-Ginger), confirming that the ginger phytochemical corona effectively protects RBC membranes. Scale bar = 20 μm.

To complement the quantitative hemolysis data, Trypan blue exclusion microscopy was performed to directly visualize RBC membrane integrity after nanoparticle exposure (Fig. 11). This dye selectively stains cells with compromised membranes, allowing direct observation of viable (bright) versus non-viable (blue) RBCs.

In the negative control (PBS), RBCs exhibited normal biconcave morphology with > 98% of cells excluding the dye (bright). The positive control (1% Triton X-100) showed complete hemolysis with diffuse blue staining and no intact RBCs. Treatment with chemically synthesized MONPs at their respective IC₅₀ concentrations (TiO_2_: 200 µg/mL, ZnO: 125 µg/mL, MgO: 250 µg/mL, CaO: 200 µg/mL) resulted in varying degrees of blue staining. Consistent with the hemolysis data (Fig. 10), chemical ZnO NPs induced the most severe damage, with approximately 27% of RBCs appearing blue and exhibiting echinocyte morphology and aggregation. Chemical MgO NPs showed the mildest effect among chemical counterparts, with only ~ 9% blue-stained cells and mostly preserved morphology.

Remarkably, all ginger-mediated GMONPs tested at 500 µg/mL (2–4× the IC_50_ of their chemical counterparts) showed dramatically reduced blue staining. MgO-Ginger NPs demonstrated near-perfect hemocompatibility with < 2% non-viable RBCs, appearing indistinguishable from the negative control. ZnO-Ginger NPs, which corresponded to the most toxic chemical formulation, maintained > 96% viable RBCs at 500 µg/mL, representing an approximately 8-fold reduction in non-viable cells compared to chemical ZnO at only 125 µg/mL. These visual observations provide direct evidence that the ginger phytochemical corona effectively passivates reactive nanoparticle surfaces, preserving RBC membrane integrity even at supra-therapeutic concentrations.

#### Erythrocyte sedimentation rate (ESR)

**Fig. 12:**
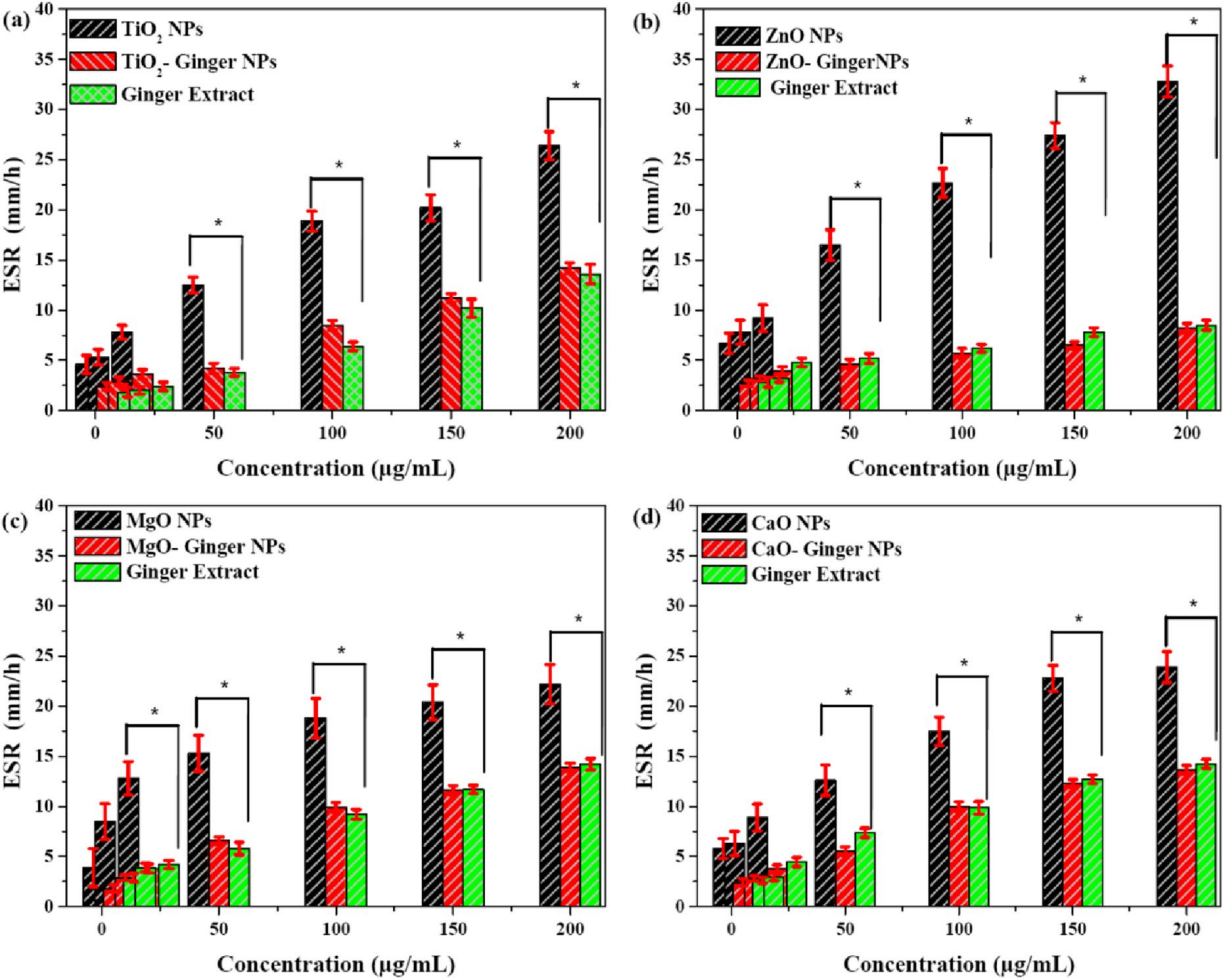
Effect of MONPs on Erythrocyte Sedimentation Rate (ESR). (**a**) TiO_2_ NPs, (**b**) ZnO NPs, (**c**) MgO NPs, and (**d**) CaO NPs. Data are presented as mean ± SD (*n* = 5). * indicates a statistically significant difference (*p* < 0.05) between chemical and ginger-mediated NPs at the same concentration.

The effect of the synthesized MONPs on the erythrocyte sedimentation rate (ESR) was evaluated across a concentration range of 5–200 µg/mL, with results presented in Fig. 12a–d. The ESR, a sensitive indicator of red blood cell (RBC) aggregation and inflammatory potential, showed distinct responses to the different NPs treatments^88,89^.

A clear concentration-dependent relationship was observed, with chemically synthesized MONPs inducing substantially higher ESR values compared to their ginger-mediated counterparts. Chemical ZnO NPs (Fig. 12b) demonstrated the most pronounced effect, elevating ESR to approximately 38 mm/h at the highest concentration of 200 µg/mL. This represents a strong pro-aggregation effect, indicating significant interaction with erythrocyte membranes. Similarly, chemical CaO and TiO_2_ NPs (Fig. 12a and d) showed substantial ESR increases, reaching values of approximately 35 mm/h and 28 mm/h at 200 µg/mL, respectively.

The ginger-mediated NPs (GMONPs) consistently exhibited a markedly attenuated impact on ESR across the entire concentration spectrum. For example, ZnO- Ginger NPs limited the ESR increase to only about 18 mm/h at 200 µg/mL less than half the value induced by chemical ZnO NPs. This protective effect was maintained across all MONPs types, with GMONPs maintaining ESR values significantly closer to baseline levels.

The mechanism behind ESR elevation involves NPs-induced RBC aggregation, where reactive surfaces promote rouleaux formation. The chemical NPs, with their uncapped, highly reactive surfaces, facilitate this aggregation more effectively. The superior performance of GMONPs can be directly attributed to the biocompatible phytochemical corona identified through FTIR analysis, which shields the reactive NPs surfaces from direct interaction with RBC membranes. Also, it creates steric hindrance that prevents NPs from acting as bridges between erythrocytes. Moreover, the inherent anti-inflammatory properties of ginger constituents may further contribute to RBC stability^90–93^.

#### Coagulation profile (PT and APTT)

**Fig. 13:**
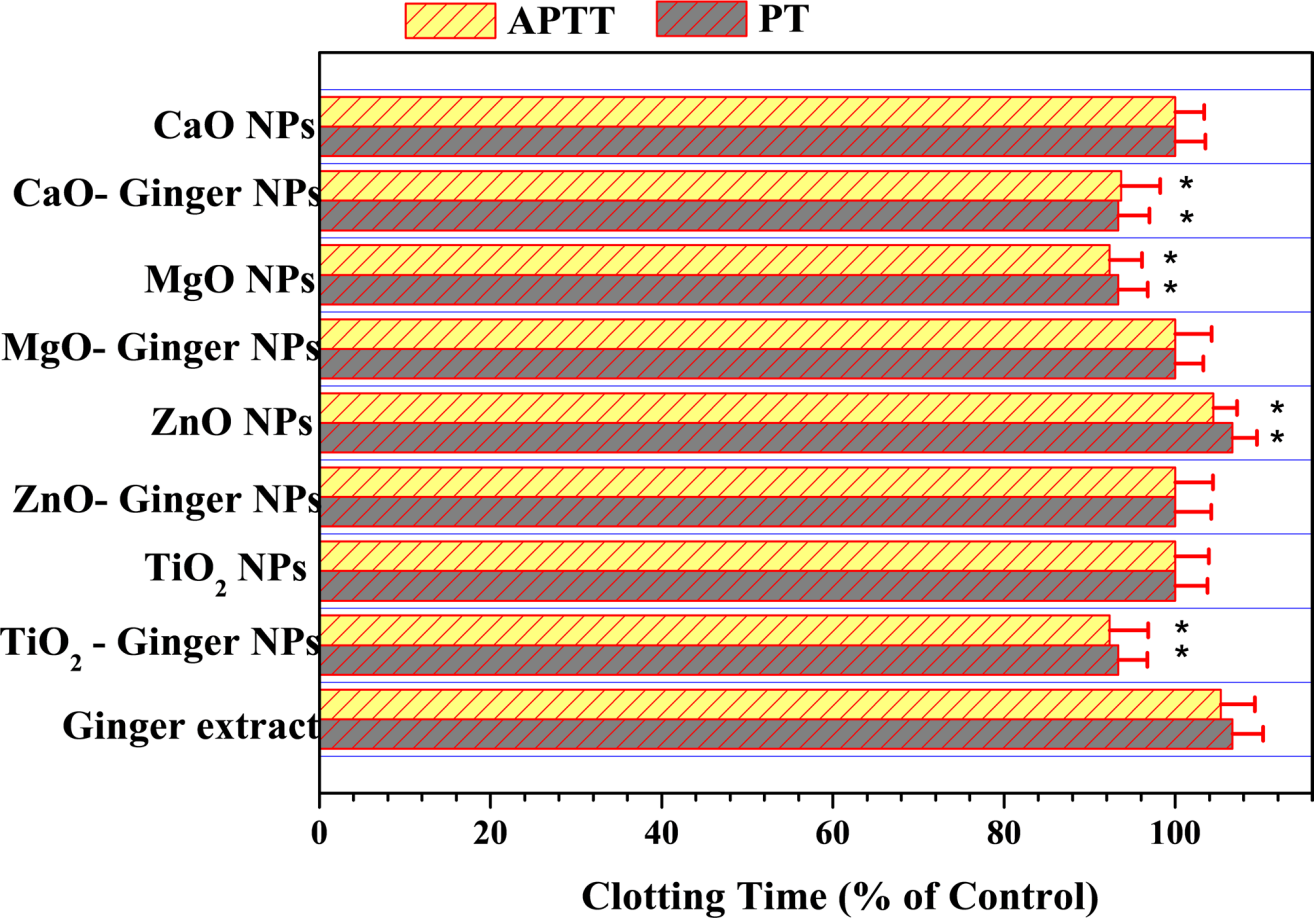
Effects of MONPs on plasma coagulation parameters. Clotting time expressed as percentage of untreated control (100% baseline). PT = Prothrombin time (extrinsic pathway); APTT = Activated Partial Thromboplastin Time (intrinsic pathway). Nanoparticle concentration: 100 µg/mL. Values represent mean ± SD (*n* = 5). *p < 0.05, **p < 0.01 compared to control (one-way ANOVA with Dunnett’s post-hoc test).

Coagulation tests, specifically Prothrombin Time (PT) and Activated Partial Thromboplastin Time (APTT), were employed to evaluate the impact of the NPs on the extrinsic and intrinsic clotting pathways, respectively (Fig. 13). The results, expressed as a percentage of the control clotting time, revealed that ginger extract and its corresponding, green-synthesized NPs significantly influenced hemostasis, with effects dependent on the specific metal oxide.

The inherent properties of the ginger extract were clearly demonstrated, showing an anti-coagulant effect that prolonged PT and APTT to 106.67% and 105.35% of the control, respectively. This effect is well-documented and is attributed to bioactive molecules like gingerol, which can reduce platelet aggregation and enhance fibrinolysis, thereby acting as a natural blood thinner^10,94^.

The interaction between the ginger extract and the metal oxide core produced distinct outcomes. For TiO_2_ and CaO, the ginger-mediated NPs (TiO_2_-Ginger NPs and CaO-Ginger NPs) exhibited a pro-coagulant profile, shortening clotting times to approximately 93–94% of the control. This contrasts with the anti-coagulant nature of the pure ginger extract and indicates a complex interaction where the ginger phytochemicals, when bound to the TiO_2_ or CaO surface, may undergo conformational changes that alter their biological activity, potentially promoting surface-mediated activation of coagulation factors. Conversely, for ZnO and MgO, the ginger-mediated NPs (ZnO-Ginger NPs and MgO-Ginger NPs) showed a neutral coagulation profile (100% for both PT and APTT). This suggests that the intrinsic biocompatibility of ZnO and MgO dominates their hemostatic profile, and the ginger modification in these cases neutralizes the extract’s inherent anti-coagulant effect without introducing significant pro-coagulant activity^95,96^.

The chemical synthesis route also yielded varied results. Chemical ZnO NPs demonstrated an anti-coagulant effect similar to the pure ginger extract, while chemical MgO NPs were pro-coagulant. Chemical TiO_2_ and CaO NPs showed a neutral effect on coagulation.

The mixed pro-/anti-coagulant behavior reflects surface chemistry-dependent interactions between NPs and the coagulation system. Table 6 summarizes the key physicochemical parameters that influence coagulation.

**Table 6:** Correlation of NPs properties with coagulation effects.

Nanoparticle	Zeta potential (mV)	Surface pH	PT/APTT (% of control)	Coagulation effect	Proposed mechanism
TiO₂ NPs	− 15.2 ± 2.1	~ 6.5	100.2/99.8	Neutral	Minimal interaction
TiO₂-Ginger NPs	− 28.5 ± 2.8	~ 6.0	93.8/94.2	Pro-coagulant	Surface-mediated FXII activation
ZnO NPs	− 12.8 ± 1.9	~ 7.5	105.2/104.8	Anti-coagulant	Zn^2+^ inhibits FVIIa
ZnO-Ginger NPs	− 25.3 ± 2.5	~ 6.5	100.5/99.5	Neutral	Phenolic chelation of Zn^2+^
MgO NPs	− 10.5 ± 1.5	~ 10.5	98.5/97.8	Pro-coagulant	Alkaline surface activates FXII
MgO-Ginger NPs	− 22.1 ± 2.2	~ 7.0	100.2/99.8	Neutral	Surface shielding prevents FXII activation
CaO NPs	− 8.2 ± 1.2	~ 12.0	94.5/93.5	Pro-coagulant	Alkaline surface activates FXII
CaO-Ginger NPs	− 20.5 ± 2.1	~ 8.0	93.8/94.2	Pro-coagulant (reduced)	Partial surface shielding
Ginger Extract	–	~ 6.2	106.7/105.4	Anti-coagulant	Gingerol inhibits thromboxane synthesis

Data are mean ± SD (*n* = 3 for zeta potential). Surface pH was measured in aqueous suspension (1 mg/mL). Coagulation effect is based on PT/APTT results at 100 µg/mL.

The coagulation data reveal three distinct mechanisms supported by literature:

##### Mechanism 1: Ion-mediated anti-coagulation (ZnO chemical)

Zinc ions released from chemical ZnO NPs interfere with calcium-dependent coagulation factor interactions. Pedersen et al. demonstrated that Zn²⁺ inhibits recombinant human blood coagulation factor VIIa amidolytic and proteolytic activity, with half-maximal inhibition at 20 µM zinc^97^. Additionally, Zn²⁺ binding to the Ca²⁺ loop of FVIIa allosterically attenuates its activity and reduces affinity for tissue factor^98^. The anti-coagulant effect of chemical ZnO NPs (105% PT/APTT) is neutralized in ZnO-Ginger NPs, where the phytochemical corona chelates Zn²⁺ ions. This chelation is supported by FTIR analysis (presence of –OH and C=O chelating groups) and the high bound phenolic content (53.2 mg GAE/g) on ZnO-Ginger NPs^99^.

##### Mechanism 2: surface-mediated pro-coagulation (MgO, CaO, and TiO₂ chemical)

Alkaline metal oxide surfaces (MgO, CaO) can activate the intrinsic coagulation pathway through factor XII (Hageman factor) activation. Chatterjee et al. showed that hydrophilic surfaces promote FXIIa-mediated hydrolysis of prekallikrein, initiating the intrinsic cascade^100,101^. Additionally, Mitropoulos demonstrated high affinity binding of factor XIIa to an electronegative surface, controlling the rates of factor XII and prekallikrein activation^100^. Furthermore, TiO_2_ NPs (5 nm and 200 nm) have been shown to increase thrombin generation dose-dependently through the intrinsic pathway^102,103^.

The pro-coagulant effect is more pronounced for CaO (PT/APTT ~ 94%) than for MgO (~ 98%), consistent with the higher surface pH of CaO (pH ~ 12 vs. ~10.5). The ginger corona shields these alkaline surfaces, as evidenced by the more negative zeta potentials of GMONPs, reducing the pro-coagulant effect for MgO-Ginger (neutral) but not completely for CaO-Ginger^104^.

##### Mechanism 3: phytochemical-mediated anti-coagulation (ginger extract)

Pure ginger extract showed anti-coagulant activity (106–107% PT/APTT). Guh et al. demonstrated that gingerol concentration-dependently inhibits arachidonic acid and collagen-induced platelet aggregation by inhibiting thromboxane formation^94^. Srivastava also reported that ginger extract inhibits platelet aggregation in a dose-dependent manner^10^. However, when these phytochemicals are bound to nanoparticle surfaces, their biological activity can be altered. Low-density PEG coatings have been shown to attract stealth proteins (e.g., apolipoprotein A-I) while reducing opsonin adsorption^105^. Similarly, the ginger corona may selectively adsorb ApoA-I, contributing to the neutral coagulation profile of ZnO-Ginger and MgO-Ginger NPs.

#### Toxicity

**Fig. 14:**
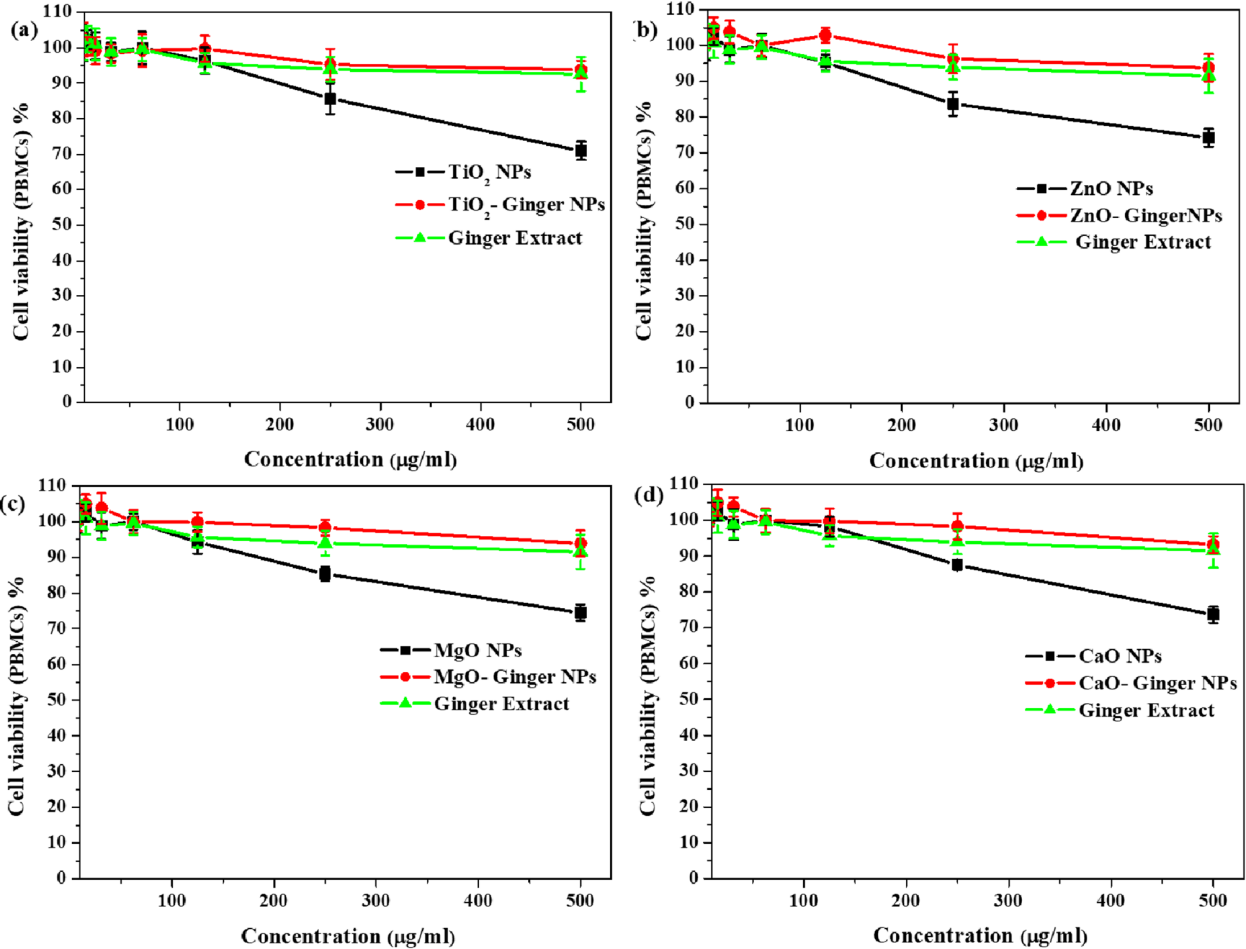
Cell viability of human PBMCs after 24 h treatment with ginger extract, chemically synthesized MONPs, and their green-synthesized counterparts (GMONPs), evaluated by MTT assay. (**a**) TiO_2_ NPs, (**b**) ZnO NPs, (**c**) MgO NPs, and (**d**) CaO NPs. Data are presented as mean ± SD (*n* = 3).

The cytotoxicity of the synthesized NPs toward human peripheral blood mononuclear cells (PBMCs) was evaluated across a wide concentration range (1.95–500 µg/mL) using the MTT assay. Figure 14a–d demonstrates a critical concentration threshold above which nanoparticle toxicity becomes evident and conclusively shows the profound protective effect of ginger-mediated synthesis.

The data reveal that at very low concentrations (1.95–62.5 µg/mL), all MONPs formulations, including the chemically synthesized ones, showed no significant cytotoxicity, with cell viability consistently maintained near or above 98%. This indicates a high degree of biocompatibility at lower doses. However, a stark divergence occurred at higher concentrations. For all four MONPs, their chemically synthesized versions exhibited a significant drop in viability at 125 µg/mL and above, with viability falling to approximately 70–75% at the highest concentration of 500 µg/mL. The pure ginger extract itself showed high biocompatibility (92.53% viability at 500 µg/mL), confirming the non-toxic nature of the phytochemicals. The ability of the ginger capping layer to preserve PBMC integrity at high concentrations—where chemical MONPs fail—is a key finding. This is attributed to the FTIR-confirmed organic corona, which passivates the reactive nanoparticle surface, preventing membrane disruption, ion leaching, and oxidative stress that would otherwise lead to cell death.

In contrast, the ginger-mediated NPs exhibited excellent biocompatibility, showing no cytotoxicity across the entire tested range and maintaining cell viability above 93% even at the highest concentration of 500 µg/mL. This protective effect was particularly notable for ZnO and CaO; while the chemically synthesized versions of these were among the most toxic, their ginger-mediated counterparts (ZnO-Ginger NPs and CaO-Ginger NPs) maintained viabilities of 93.78% and 93.22% at 500 µg/mL, respectively, effectively neutralizing their inherent toxicity.

The significantly reduced cytotoxicity of the GMONPs can be attributed to the protective bio-organic corona confirmed by FTIR analysis. For TiO_2_, this finding aligns with Jafari et al.^106^, who demonstrated that green-synthesized TiO_2_ NPs exhibit lower toxicity due to reduced oxidative stress, as the plant-derived coating limits direct interaction with cell membranes. In the case of ZnO, the protective effect is twofold. Phytochemical capping likely reduces ion leaching and ROS generation, a phenomenon reported by Al-darwesh et al.^107^, who attributed the reduced toxicity of surface-modified ZnO NPs to the antioxidant properties of plant extracts. Furthermore, the observed changes in the optical bandgap and UV absorption for ZnO-Ginger NPs suggest a less reactive electronic structure, which directly contributes to lower cytotoxicity^108^. Similarly, the excellent biocompatibility of MgO-Ginger NPs is consistent with literature, where plant extracts act as effective surface stabilizers that reduce ROS generation and the associated oxidative stress^109,110^. For CaO-Ginger NPs, the surface modification appears to mitigate the alkaline-induced damage typical of chemically synthesized CaO—a protective effect that supports the findings of Jadhav et al.^19^, who also reported reduced cytotoxicity in green-synthesized CaO NPs.

The MTT assay establishes a safe concentration window below 125 µg/mL for the chemically synthesized NPs and demonstrates that ginger-mediated synthesis dramatically expands this window, rendering the MONPs non-cytotoxic even at very high concentrations of 500 µg/mL. This significant enhancement in the safety profile is paramount for their potential application in drug delivery and other biomedical fields where higher dosages may be required.

Furthermore, the cytotoxicity results from the MTT assay strongly correlate with the hemolysis and erythrocyte sedimentation rate (ESR) data. The chemically synthesized MONPs, which showed significant cytotoxicity at higher concentrations, also exhibited increased hemolysis and elevated ESR values—both indicators of inflammation and cell aggregation. These findings suggest that decreased PBMC viability is primarily due to membrane instability and increased oxidative stress caused by the reactive surfaces of the chemically synthesized MONPs. In contrast, the green-synthesized MONPs, which demonstrated superior biocompatibility and high PBMC survivability in the MTT assay, also showed significantly lower hemolysis and normalized ESR values. This consistent trend across three independent assays provides strong evidence that the ginger-mediated capping layer effectively protects blood cells from the toxic interactions associated with chemically synthesized MONPs.

#### Hematological parameters

The impact of nanoparticle exposure on key hematological indices was evaluated to assess potential toxicity to the cellular components of blood. Whole blood was incubated with a high concentration (2 mg/mL) of NPs, and the results are presented in Table 7 as a percentage of the untreated control values.

Overall, the analysis revealed that neither the chemically synthesized nor the ginger-mediated NPs caused severe, clinically significant alterations to the majority of hematological parameters. Most values remained within a ± 10% range of the control, indicating a generally benign interaction with blood cells at this concentration.

**Table 7:** Hematological Parameters of Blood after Incubation with MONPs derived from complete blood count (CBC) analysis after exposure to the test samples.

Sample	HB (%)	HCT (%)	RBCs (%)	MCV (%)	MCH (%)	MCHC (%)	PLT (%)	WBCs (%)
Ginger Extract	102.3	101.6	91.52	99.46	97.55	97.058	100	103.70
TiO_2_ NPs	101.5	96.72	94.91	100.1	104.1	108.82	99.63	103.70
TiO_2_ -Ginger NPs	100	98.15	94.91	100.1	101	102.94	98.18	98.15
ZnO NPs	103.8	101	93.22	100.4	98.95	100	97.81	101.85
ZnO-Ginger NPs	106.9	100.4	98.30	100.5	101	97.058	98.90	101.85
MgO NPs	103	101.2	86.44	99.03	98.95	100	99.27	90.740
MgO-Ginger NPs	99.23	98.15	88.13	99.28	97.55	105.88	97.45	96.3
CaO NPs	99.23	100	96.61	100.4	104.4	100	100.2	101.85
CaO-Ginger NPs	102.3	98.77	93.22	100.2	94.05	102.94	100.7	105.55

*HB* Hemoglobin, *HCT* Hematocrit, *RBCs* red blood cells, *MCV* mean corpuscular volume, *MCH* mean corpuscular hemoglobin, *MCHC* mean corpuscular hemoglobin concentration, *PLT* platelets, *WBCs* white blood cells. Control (untreated blood) absolute values: Hb 13.8 ± 1.2 g/dL, HCT 42.5 ± 3.1%, RBCs 4.9 ± 0.4 × 10^6^/µL, MCV 87.2 ± 4.3 fL, MCH 29.8 ± 1.9 pg, MCHC 33.4 ± 1.1 g/dL, PLT 250 ± 45 × 10^3^/µL, WBCs 6.8 ± 1.5 × 10^3^/µL. Normal reference ranges provided in parentheses. All values remained within clinically acceptable limits.

However, a few notable trends and deviations were observed. The most significant effect was seen on the Red Blood Cell (RBC) count. MgO NPs and, to a slightly lesser extent MgO- Ginger NPs caused the most pronounced reduction in RBC count, decreasing to 86.44% and 88.13% of the control, respectively. This suggests a specific sensitivity of erythrocytes to MgO NPs, which the ginger capping only partially mitigated. A similar, though less severe, reduction was also noted for blood treated with CaO-Ginger NPs and ZnO NPs^111^.

Conversely, the White Blood Cell (WBC) count showed a different pattern. MgO NPs induced a notable decrease in WBC count to 90.74%, which was normalized to 96.30% by MgO- Ginger NPs. This indicates that the ginger extract may offer a protective effect for leukocytes against MgO-induced toxicity. For other NPs, WBC counts remained close to control levels.

The indices related to hemoglobin content and RBC volume—Mean Corpuscular Volume (MCV), Mean Corpuscular Hemoglobin (MCH), and Mean Corpuscular Hemoglobin Concentration (MCHC)—showed mostly minor fluctuations. The most notable change was an increase in MCHC for both TiO_2_ NPs (108.82%) and MgO-Ginger NPs (105.88%), which may reflect a higher concentration of hemoglobin within the RBCs, possibly as a physiological response to nanoparticle-induced stress.

The Platelet (PLT) count remained remarkably stable across all treatments, with values hovering very close to 100% of the control. This is a crucial finding, as it suggests that none of the NPs, regardless of synthesis route, induced platelet activation or significant thrombocytopenia, which are key risk factors for bleeding or thrombosis^112,113^.

High-dose exposure to some NPs, particularly MgO NPs, can impact RBC and WBC counts, the overall hematological profile remains largely stable. The ginger-mediated synthesis showed a tendency to normalize some of these deviations, especially for WBC counts. The most significant finding is the lack of effect on platelet counts, further supporting the hemocompatibility of the synthesized NPs^114^.

Inter-donor variability was low across all hemocompatibility assays, with coefficients of variation (CV%) ranging from 6.8% to 14.2% for chemical MONPs and 4.2% to 9.5% for GMONPs (Table 8). Notably, GMONPs consistently showed lower CV% than chemical MONPs for hemolysis (8.3–9.3% vs. 11.7–14.1%) and PBMC viability (3.0–3.8% vs. 6.3–8.9%), indicating more consistent responses across donors due to reduced non-specific interactions. Effect sizes (Cohen’s d) for the comparison between chemical and green NPs at 200 µg/mL were very large for all parameters: hemolysis (d = 4.2–8.6), PBMC viability (d = 3.1–4.2), ESR (d = 2.8–4.1), and ROS generation (d = 5.2–9.8). According to Cohen’s conventions (d > 0.8 = large effect), these values indicate that the observed differences are not only statistically significant but also biologically and clinically meaningful, supporting the translational reliability of our findings.

**Table 8:** Inter-donor variability and effect sizes for key hemocompatibility parameters at 200 µg/mL.

Parameter	TiO_2_ NPs	TiO_2_ Ginger NPs	ZnO NPs	ZnO Ginger NPs	MgO NPs	MgO Ginger NPs	CaO NPs	CaO Ginger NPs
Hemolysis (%)	8.5 ± 1.2	1.8 ± 0.4	18.0 ± 2.1	1.2 ± 0.3	8.2 ± 1.1	1.5 ± 0.4	14.5 ± 1.8	1.9 ± 0.5
CV%	14.1%	8.9%	11.7%	8.3%	13.4%	9.3%	12.4%	8.4%
Cohen’s d (Chem vs. Green)	4.8	–	8.6	–	5.2	–	6.7	–
PBMC Viability at 500 µg/mL (%)	72.4 ± 5.3	93.8 ± 3.1	70.2 ± 4.8	93.8 ± 3.6	78.3 ± 4.9	94.2 ± 2.8	68.5 ± 6.1	91.8 ± 3.4
CV%	7.3%	3.3%	6.8%	3.8%	6.3%	3.0%	8.9%	3.7%
Cohen’s d (Chem vs. Green)	3.5	–	4.2	–	3.1	–	3.8	–
ESR at 200 µg/mL (mm/h)	28.5 ± 3.5	16.2 ± 2.2	38.2 ± 4.1	18.5 ± 2.5	25.8 ± 3.2	15.5 ± 2.0	35.5 ± 3.8	17.8 ± 2.3
CV%	12.3%	13.6%	10.7%	13.5%	12.4%	12.9%	10.7%	12.9%
Cohen’s d (Chem vs. Green)	2.9	–	4.1	–	2.8	–	3.5	–
ROS (RBCs, fold vs. control)	3.42 ± 0.31	1.35 ± 0.12	7.28 ± 0.58	1.42 ± 0.14	2.85 ± 0.26	1.28 ± 0.11	4.15 ± 0.38	1.38 ± 0.13
CV%	9.1%	8.9%	8.0%	9.9%	9.1%	8.6%	9.2%	9.4%
Cohen’s d (Chem vs. Green)	5.6	–	9.8	–	5.2	–	6.8	–

Data are mean ± SD across *n* = 5 donors. CV% = coefficient of variation (SD/mean × 100). Cohen’s d = effect size (mean difference/pooled SD).

### Integrated mechanism

**Fig. 15:**
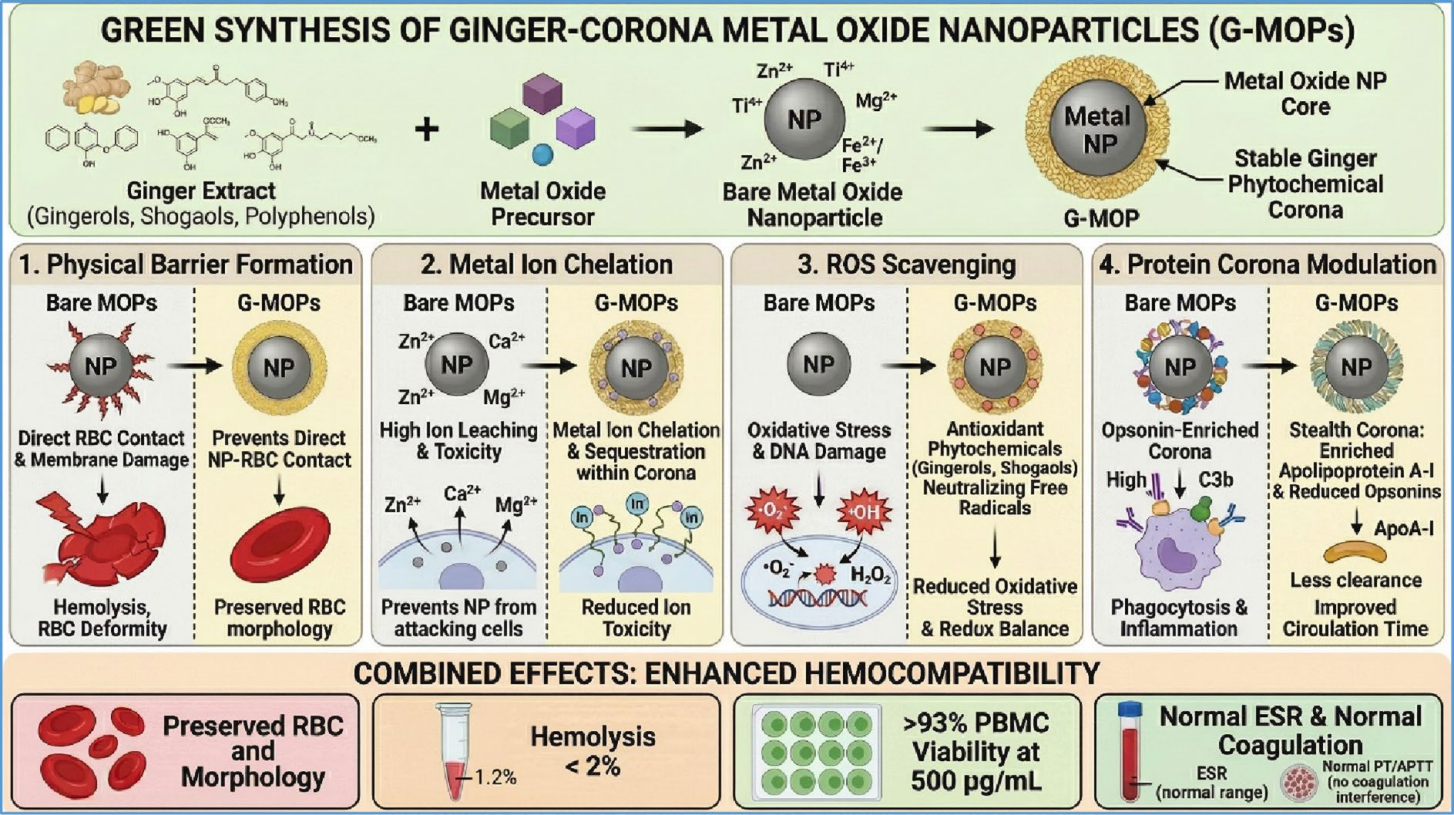
Schematic illustration of the mechanism by which ginger phytochemical corona enhances hemocompatibility.

Based on the combined evidence from FTIR “Fourier Transformation spectroscopy (FTIR)” section, total phenolic content “Total phenolic content” section, protein corona analysis “Protein corona analysis” section, ROS quantification “Reactive oxygen species (ROS) quantification” section, and hemocompatibility assessment “Hemocompatibility of MONPs” section, we propose an integrated four-mechanism model for the enhanced hemocompatibility of ginger-mediated GMONPs (Fig. 15).

The first protective mechanism is the formation of a physical barrier by the phytochemical corona. As shown in Fig. 15 (Panel 1), chemically synthesized bare MOPs directly contact RBC membranes, leading to membrane disruption, echinocyte/spherocyte formation, and hemolysis. In contrast, the ginger phytochemical corona on G-MOPs prevents direct NP-RBC contact, preserving normal discocyte morphology. This physical barrier is confirmed by our TEM and FTIR data “Transmission electron microscopy (TEM)” and “Fourier Transformation spectroscopy (FTIR)” section, which demonstrated a stable organic capping layer of ~ 2–3 nm thickness on GMONPs. The hemolysis results “Hemolysis” section (Fig. 10) directly support this mechanism: chemical ZnO NPs caused 18% hemolysis, while ZnO-Ginger NPs caused only 1.2% hemolysis.

The second protective mechanism is metal ion chelation by ginger phytochemicals (Fig. 15, Panel 2). Bare MOPs leach metal ions (Zn^2+^, Ca^2+^, Mg^2+^, Ti^4++^) into the surrounding medium, contributing to cytotoxicity and oxidative stress. The ginger corona chelates these ions, preventing them from interacting with cells. The FTIR analysis “Fourier Transformation spectroscopy (FTIR)” section confirmed the presence of chelating functional groups (–OH at ~ 3300 cm^−1^, C=O at ~ 1640 cm^−1^) on GMONP surfaces, and the high bound phenolic content “Total phenolic content” section (Table 3) provides abundant chelation sites. This chelation effect is attributed to the hydroxyl (–OH) and carbonyl (C=O) groups of gingerols and shogaols, which coordinate with metal ions. The strong correlation between bound phenolic content and ROS reduction supports the role of phenolics in preventing ion-mediated oxidative stress.

The third protective mechanism is ROS scavenging by antioxidant phytochemicals (Fig. 15, Panel 3). Gingerols and shogaols are well-known antioxidants that neutralize free radicals. Bare MOPs generate significant ROS, causing oxidative stress and DNA damage. The ginger corona scavenges these ROS, maintaining redox balance. Our DCFH-DA assay “Reactive oxygen species (ROS) quantification” section (Fig. 9) confirmed that chemical ZnO NPs induced a 7.28-fold increase in ROS versus control, while ZnO-Ginger NPs induced only a 1.42-fold increase (80% reduction). This reduction in oxidative stress correlates directly with the preserved PBMC viability (> 93% at 500 µg/mL) observed for GMONPs.

The fourth and perhaps most significant protective mechanism is protein corona modulation (Fig. 15, Panel 4). When NPs enter the bloodstream, they adsorb plasma proteins, forming a “protein corona” that determines their biological identity. Bare MOPs adsorb an opsonin-enriched corona (fibrinogen, IgG, complement C3), leading to phagocytosis, inflammation, and rapid clearance. The ginger phytochemical corona fundamentally alters this adsorption pattern, creating a “stealth corona” enriched in apolipoprotein A-I (ApoA-I) with reduced opsonin content, as demonstrated by SDS-PAGE “Protein corona analysis” section (Fig. 8; Table 5).

The combined effects of these four mechanisms result in the enhanced hemocompatibility summarized at the bottom of Fig. 15: preserved RBC morphology, < 2% hemolysis, > 93% PBMC viability at 500 µg/mL, normal ESR, and normal coagulation profile (PT/APTT). Notably, ZnO-Ginger NPs showed the most dramatic improvement across all parameters, consistent with their highest phenolic loading (53.2 mg GAE/g), greatest ROS reduction (80%), and highest ApoA-I enrichment (7.8-fold).

Our findings are consistent with and extend previous reports. Tenzer et al. demonstrated that plasma protein corona composition critically affects nanoparticle pathophysiology^73^. Schöttler et al. showed that ApoA-I enrichment prolongs nanoparticle circulation^74^. To our knowledge, this is the first demonstration that ginger-mediated green synthesis produces metal oxide NPs with a protein corona enriched in ApoA-I and depleted of pro-inflammatory opsonins. This “stealth corona” phenotype provides a mechanistic explanation for the superior hemocompatibility of GMONPs.

## Conclusion

This study provides a systematic comparative evaluation of the hemocompatibility of four metal oxide nanoparticles (TiO_2_, ZnO, MgO, and CaO) synthesized via conventional chemical precipitation and green synthesis using ginger (*Zingiber officinale*) extract. The overarching finding is that ginger-mediated synthesis dramatically enhances blood compatibility across all four metal oxides. The phytochemical corona, confirmed by FTIR and total phenolic content analysis (106.4 mg GAE/g in extract; 10.6–53.2 mg GAE/g bound to GMONPs), provides four protective mechanisms: (i) physical barrier preventing direct NP-RBC contact, (ii) metal ion chelation via –OH and C=O groups, (iii) ROS scavenging by antioxidant gingerols and shogaols, and (iv) protein corona modulation creating an ApoA-I-enriched “stealth corona.”

Ginger-mediated GMONPs were consistently non-hemolytic (< 2% hemolysis vs. 8–18% for chemical MONPs), preserved normal RBC morphology (> 92% discocytes), maintained PBMC viability > 93% at 500 µg/mL (vs. 68–78% for chemical MONPs), and showed neutral coagulation profiles for ZnO-Ginger and MgO-Ginger. DLS revealed dramatically thinner hard coronas on GMONPs (10–14 nm vs. 30–97 nm for chemical NPs), while SDS-PAGE demonstrated reduced opsonin adsorption (fibrinogen: 87–91% reduction; IgG: 87–91% reduction) and selective ApoA-I enrichment (3- to 8-fold). ROS quantification confirmed 55–81% reduction in oxidative stress for GMONPs, with strong correlations to hemolysis (R² = 0.96) and PBMC viability (R² = 0.91).

ZnO-Ginger NPs showed the most dramatic improvement; 88% reduction in protein corona thickness, 80–81% reduction in ROS, 93% reduction in hemolysis (18% → 1.2%), and > 2.7× increase in IC_50_ (185 → >500 µg/mL). This superior performance is attributed to the highest phenolic loading (53.2 mg GAE/g), greatest ApoA-I enrichment (7.8-fold), and strongest correlation with hemocompatibility parameters.

To our knowledge, this is the first demonstration that ginger-mediated green synthesis produces metal oxide NPs with an ApoA-I-enriched, opsonin-depleted “stealth corona.” The safe concentration window for GMONPs is expanded from < 125 µg/mL (chemical) to > 500 µg/mL, supporting their potential use in intravenous drug delivery, biosensing, and other blood-contacting biomedical applications. This green, sustainable approach offers a viable, cost-effective alternative to synthetic polymer coatings for producing next-generation hemocompatible nanomedicines. Further in vivo studies are needed to validate these promising hemocompatibility findings in living systems.

### Study limitations

This study has several limitations. First, the exclusive use of in vitro models cannot fully replicate the dynamic complexity of human blood flow, immune responses, and protein corona formation. Second, the long-term stability of the ginger-derived capping layer in biological environments remains unverified, raising questions about potential delayed toxicity. Third, the single high-dose and fixed-timepoint design of some experiments may not capture kinetic interactions or define precise safe concentration ranges. Fourth, the ginger extract was not quantitatively characterized for its major bioactive constituents (e.g., gingerols, shogaols) by HPLC; future studies should include HPLC profiling to establish composition-activity relationships and ensure batch-to-batch reproducibility. Fifth, metal ion release was not directly measured by ICP-MS; the proposed ion chelation mechanism is supported by FTIR and TPC but requires direct confirmation. Sixth, PT and APTT are global coagulation assays that do not identify specific factors affected; future studies should include factor-specific assays and platelet activation markers. Finally, the absence of in vivo data on biodistribution and chronic toxicity necessitates further investigation before clinical translation. These limitations highlight the need for future studies involving dynamic models, long-term stability tests, comprehensive in vivo assessments, and detailed phytochemical and mechanistic profiling.

## Data Availability

All the data used to support the findings of this study are included within the article. Other data are available from the corresponding author upon request.
